# Size dependence of phase transitions in aerosol nanoparticles

**DOI:** 10.1038/ncomms6923

**Published:** 2015-01-14

**Authors:** Yafang Cheng, Hang Su, Thomas Koop, Eugene Mikhailov, Ulrich Pöschl

**Affiliations:** 1 Multiphase Chemistry Department, Max Planck Institute for Chemistry, 55128 Mainz, Germany; 2 Faculty of Chemistry, Bielefeld University, 33615 Bielefeld, Germany; 3 Institute of Physics, St. Petersburg State University, 198904 St. Petersburg, Russia

## Abstract

Phase transitions of nanoparticles are of fundamental importance in atmospheric sciences, but current understanding is insufficient to explain observations at the nano-scale. In particular, discrepancies exist between observations and model predictions of deliquescence and efflorescence transitions and the hygroscopic growth of salt nanoparticles. Here we show that these discrepancies can be resolved by consideration of particle size effects with consistent thermodynamic data. We present a new method for the determination of water and solute activities and interfacial energies in highly supersaturated aqueous solution droplets (Differential Köhler Analysis). Our analysis reveals that particle size can strongly alter the characteristic concentration of phase separation in mixed systems, resembling the influence of temperature. Owing to similar effects, atmospheric secondary organic aerosol particles at room temperature are expected to be always liquid at diameters below ~20 nm. We thus propose and demonstrate that particle size should be included as an additional dimension in the equilibrium phase diagram of aerosol nanoparticles.

## Abstract (web summary)

Although phase transitions are fundamental for understanding aerosol particles, current models are insufficient to explain observations at the nanoscale. Here, the authors present a method for investigating droplets, suggesting particle size is a key determinant in the phase diagram of nanoparticles.

## Graphical abstract

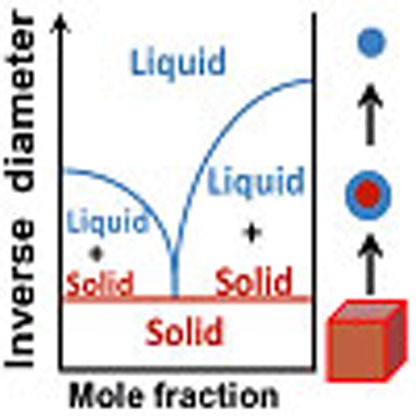

The phase state of aerosol particles is a key determinant of their physico-chemical interactions, climate and health effects^1^,^2^,^3^,^4^. Chemical composition, relative humidity and temperature have been recognized as the main factors controlling aerosol phase state^1^,^2^,^5^, but they are insufficient to explain observations for nanometer-sized particles, even for well-studied inorganic reference substances^3^,^6^,^7^,^8^,^9^,^10^, such as ammonium sulphate (AS).

Progress in resolving these discrepancies has been hampered by a lack of reliable thermodynamic data, such as water and solute activities and interfacial energies. This lack of data exists because nanodroplets can become more highly supersaturated (with respect to a crystal) when compared with bulk solution samples, but traditional measurement techniques and data, especially the liquid-vapour interfacial energy (*σ*_lv_, also called surface tension), are mostly limited to sub-saturated and saturated solutions. The existing measurement techniques for *σ*_lv_ often require touching the solution by needles or other intermediates, such as a plate or capillary tube^11^. Solute crystallization induced by heterogeneous nucleation can take place in supersaturated solution on the contact surface, which makes the precise measurements difficult. Measurement of *σ*_lv_ using the vibration modes of a magnetically levitated drop could avoid the contact but is limited to supermicrometre particles owning to the stability in the magnetic trap^12^. Measurements of water activities (*a*_w_) have been extended to supersaturated range by Electrodynamic Balance (EDB) methods^13^,^14^ (for example, up to ~30 mol kg^−1^ for AS), but it is not applicable for the nanosize range either.

To overcome these limitations, we develop a new method for the determination of these specific thermodynamic parameters, closing the gap between experimental and modelled results. Further analysis reveals that particle size can strongly alter the phase separation process in mixed systems. We thus propose and demonstrate that particle size should be included as an additional dimension in the equilibrium phase diagram of aerosol nanoparticles.

## Results

### Thermodynamic properties of supersaturated nanodroplets

**Figure 1:**
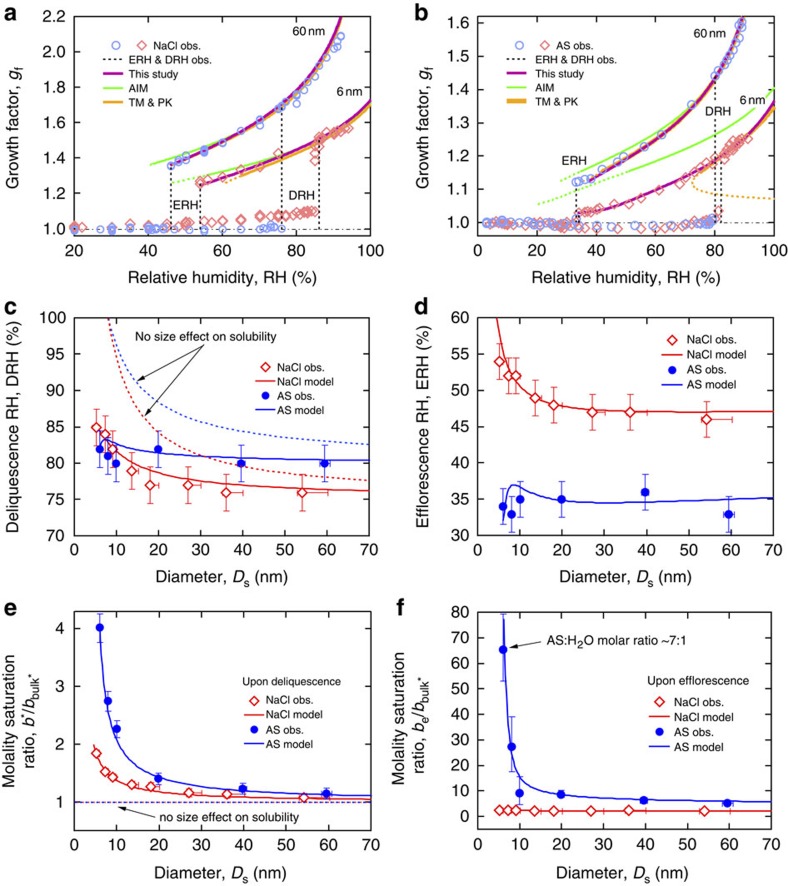
Hygroscopic growth, deliquescence and efflorescence of nanoparticles. (**a**,**b**) Hygroscopic growth curves of sodium chloride (NaCl) and ammonium sulphate (AS). Growth factor data are taken from HTDMA experiments^8^,^9^ for particles with dry diameters of 60 nm (blue circle) and 6 nm (red diamond). Köhler model curves are based on different thermodynamic data sets: in this study, purple line with DKA (Differential Köhler Analysis) derived data for AS and modified TM & SP (Tang-Munkelwitz^35^,^36^ and Seinfeld-Pandis^37^) for NaCl; AIM (Aerosol Inorganics Model^38^), green line; TM & PK (Tang-Munkelwitz^35^,^36^ and Pruppacher-Klett^39^) as in Biskos *et al.*^8^,^9^, orange line (Supplementary Note 3). Dashed lines indicate extrapolation beyond validated concentration range. Size-dependent threshold values of relative humidity and solute saturation ratio on deliquescence (**c**,**e**) and efflorescence (**d**,**f**). Saturation ratios refer to bulk water solubility expressed in solute molality (*b*_bulk_*). Model curves were obtained by combination of the Köhler equation with the Ostwald–Freundlich equation for deliquescence and with classical nucleation theory for efflorescence. Dashed lines indicate model predictions without consideration of size-dependent solubility. Error bars are estimated by considering measurement uncertainties.

According to theoretical predictions, AS nanoparticles are expected to show a strong size dependence of deliquescence relative humidity (DRH) similar to sodium chloride (NaCl), with differences up to ~10% RH between particles with dry diameters of 6 and 60 nm (refs 6, 8; Fig. 1a). Experimental data, however, reveal a much weaker size dependence for AS, that is, similar DRH values for 6-nm and 60-nm particles^9^ (Fig. 1b). Moreover, model calculations based on previously available thermodynamic data agree reasonably well with the hygroscopic growth observed for both 60-nm and 6-nm NaCl particles and also for 60-nm AS particles, but deviate strongly for 6-nm AS particles as shown in Fig. 1a,b. Further investigations of these discrepancies, however, were impeded by a lack of reliable thermodynamic data.

**Figure 2:**
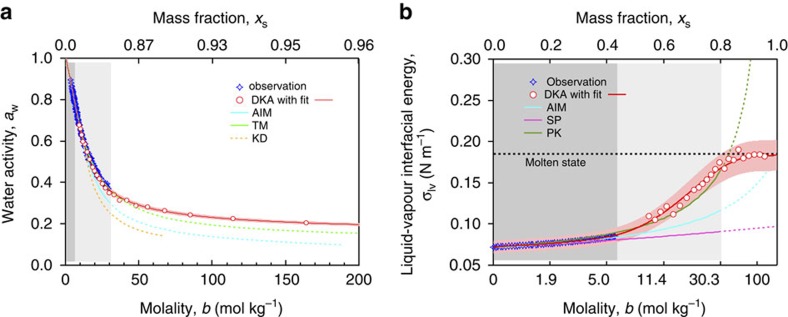
Concentration-dependent thermodynamic properties of ammonium sulphate solution. (**a**,**b**) Water activity (*a*_w_, on a mole fraction basis) and liquid-vapour interfacial energy (*σ*_lv_) are plotted against solute molality (*b*) and mass fraction (*x*_s_). The DKA-derived *a*_w_ and *σ*_lv_ (red open circles) are compared with observations (blue stars)^39^,^40^, AIM (Aerosol Inorganic Model^38^, blue line), and other parameterizations for *a*_w_ (TM=Tang-Munkelwitz^35^, green line; KD=Kreidenweis^41^, orange line) and *σ*_lv_ (PK=Pruppacher-Klett^39^, dark green line; SP=Seinfeld-Pandis^37^, purple line) (Supplementary Note 3). Dashed lines indicate extrapolation beyond validated concentration range. The equations for the best fit of DKA results (red lines) can be found in Supplementary Table 1. Red shaded areas indicate the uncertainties in the DKA retrieval, estimated by Monte Carlo analyses (Supplementary Note 1). The dark and light grey shaded areas mark the sub-saturated and saturated concentration with respect to bulk solution and supersaturated concentration before efflorescence of supermicrometre droplets, respectively. The white area marks the highly supersaturated concentration where the *a*_w_ data are not available in the literature.

In this study, a new method, termed Differential Köhler Analysis (DKA), is developed for the determination of thermodynamic properties from hygroscopic growth measurement data of aerosolized nanodroplets. Differential application of the classical Köhler equation to aerosol particles with the same hygroscopic growth factor but different dry diameters enables the simultaneous determination of *a*_w_ and *σ*_lv_ as a function of solute concentration (see Methods and Supplementary Note 1). By applying the DKA approach to high precision measurement data from hygroscopic tandem differential mobility analyser (HTDMA) experiments^9^ (see Methods), we obtained consistent and robust estimates for *a*_w_ and *σ*_lv_ of aqueous AS nanoparticles with molalities up to *b* ~160 mol kg^−1^ (solute mass fractions up to *x*_s_ ~0.96) (Fig. 2). The DKA results agree well with literature data in the previously accessible range of concentrations and enable accurate model calculations of the hygroscopic growth of highly concentrated AS nanoparticles (purple line in Fig. 1b); for DKA results of NaCl nanoparticles see Supplementary Fig. 1.

### Measured and predicted phase transitions and equilibria

For model predictions of the DRH and ERH (efflorescence relative humidity), we calculated solute activity (*a*_s_) and obtained the interfacial energy of the salt in its saturated aqueous solution upon deliquescence (*σ*_sl_) and the interfacial energy of salt embryos (*γ*_*s*l_) in highly supersaturated aqueous solution upon efflorescence based on the DKA-derived *a*_w_ and *σ*_lv_ (Supplementary Notes 2,3). With this approach, we are able to determine *σ*_*s*l_ and *γ*_*s*l_ at one particular diameter and then apply them for predictions over the entire size range of 6–60 nm (see Methods). As shown in Fig. 1c,d, the DKA-based thermodynamic data allow quantifying and reconciling the contrasting size dependencies in the relative humidity thresholds of the deliquescence and efflorescence transitions for both AS and NaCl nanoparticles. The experimental DRH values observed for NaCl and AS nanoparticles are in a good agreement with our Köhler-Ostwald-Freundlich model^15^ using DKA data (solid lines in Fig. 1c) but not with the modelling approach neglecting the effect of particle size on solubility (dashed lines in Fig. 1c). The solute molality on deliquescence (b*) represents the size-dependent water solubility of nanoparticles. It is much higher than the solubility of bulk material (*b*_bulk_*) and agrees well with the Ostwald-Freundlich model prediction (Fig. 1e). Owing to a lack of reliable *a*_s_ and *σ*_*s*l_ data, previous studies failed to reproduce the size-dependent solubility of AS, and therefore, were not able to explain the different size dependence of DRH for AS and NaCl^6^,^7^.

**Figure 3:**
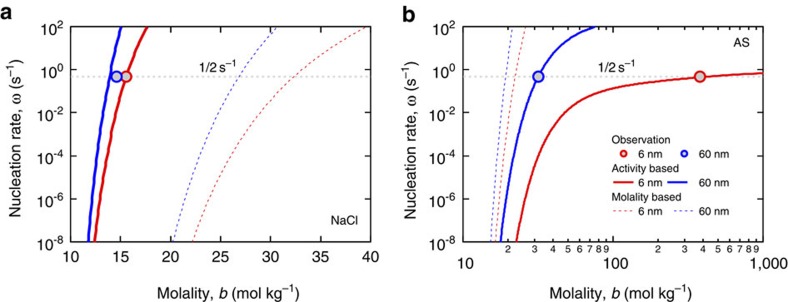
Homogeneous nucleation rate as a function of solute molality. (**a**) Sodium chloride (NaCl) and (**b**) ammonium sulphate (AS). The nucleation rates (*ω*) are calculated from solute activity (thick lines) and concentration (thin dashed lines) at selected particle sizes (6 and 60 nm). Grey dashed line corresponds to *ω* of 0.5 s^−1^, at which at least one nucleation event is triggered during the expected induction time interval (*t*_i_ and *ωt*_*i*_=1). Here, *t*_i_ is estimated as 2 s according to the residence time in the HTDMA experiments^9^. The circles represent the observed nucleation (efflorescence) concentrations.

The observed ERH values also agree very well with our predictions using DKA data (Fig. 1d) in a model combining the Köhler equation and classical nucleation theory^16^,^17^ (see Methods). The solute molality upon efflorescence (*b*_e_) represents the threshold concentration triggering solute nucleation and liquid–solid phase separation. It is not sensitive to particle size for NaCl, where a 30% increase in *b*_e_ was sufficient to maintain the same nucleation rate despite a 1,000-fold volume reduction between 60-nm and 6-nm particles (Fig. 3a and red diamonds in Fig. 1f). In contrast, an about 12-fold increase in *b*_e_ was required to trigger the nucleation and phase separation when going from 60-nm to 6-nm AS particles (Fig. 3b and blue circles in Fig. 1f). The different behaviour of NaCl and AS can be attributed largely to different relations between solute activity and concentration, that is, for the same change of activity, a stronger increase in molality is required for AS than for NaCl (Supplementary Fig. 2).

It has been shown that water activity rather than molality is the determinant for the homogenous nucleation of ice in solutions^18^. Similarly, our results demonstrate that solute nucleation and phase separation are determined by solute activity, implying that the non-ideality of solution properties become vital for the calculation of nucleation rates and understanding of phase transitions, especially for nanodroplets (Fig. 3). Indeed, the weaker size dependence of the threshold relative humidity (DRH, ERH) of AS is a consequence of its stronger size dependence of the threshold solute concentration (b*, *b*_e_) compared with NaCl (Supplementary Fig. 3).

The existence of metastable AS droplets with molalities up to ~380 mol kg^−1^ (AS:H_2_O molar ratio ~7:1) upon efflorescence (Fig. 1f) provides new insight on the phase state of salt nanoparticles. Such a high solute-to-solvent ratio implies that the droplet is no longer a typical aqueous solution, but may be regarded as a ‘molten’ salt particle with a few water molecules as a ‘solute’ or ‘impurity’ (here ‘molten’ does not imply a high melting point). By further reducing particle size, we expect an increase in the AS mole fraction, such that the particle ultimately becomes a pure molten salt at ambient temperature. This is also supported by the fact that the interfacial energy *σ*_lv_ retrieved here at a temperature *T*=298 K approaches a value of 0.182 N m^−1^ (Fig. 2b), which is consistent with the value of 0.185 N m^−1^ predicted for a molten AS at the same temperature^19^.

### Size effects on phase transitions

**Figure 4:**
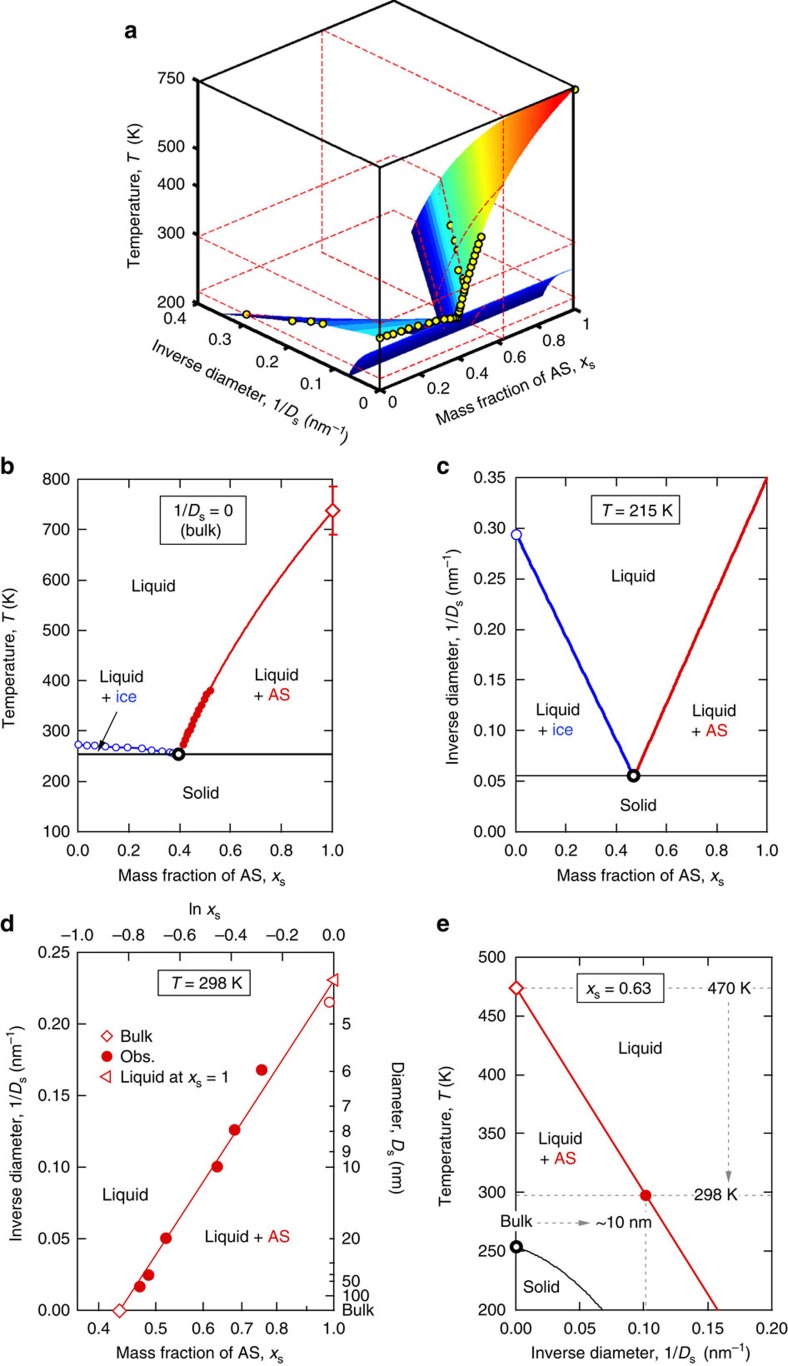
Liquid–solid equilibrium phase diagrams for the ammonium sulphate—water system. (**a**) 3D phase diagrams in the coordinates of inverse diameter 1/*D*_s_, temperature *T* and ammonium sulphate (AS) mass fraction *x*_s_. The solid circles represent the available data of bulk phase diagram of aqueous AS solution^42^, size-dependent melting temperature of ice^23^ and solubility of AS. The surfaces, coloured by the corresponding temperature, are estimated from polynomial fitting, showing the equilibrium between liquid and crystalline phases. The dashed planes illustrate the following slices. (**b**) The *T*-*x*_s_ phase diagram for bulk solution. The temperature-dependent phase separation of aqueous AS bulk solution (red line) is constrained by the melting temperature of AS at *x*_s_=1 (open diamond). The error bar shows the uncertainty of AS melting temperature according to close-cell measurements^43^. The *D*_s_^−1^-*x*_s_ phase diagram at 215 K (**c**) and at 298 K (**d**). In (**d**), the solid circles represent the observations for nano-sized droplets. The open diamond is the bulk solubility and the open circle is predicted by the DKA-constrained solute activity. The critical melting diameter (open triangle) is obtained by linear extrapolation to *x*_s_=1 (molten state). (**e**) The *T*-*D*_s_^−1^ phase diagram at *x*_s_=0.63. The black open circle represents the eutectic point for bulk solution (also in **b** and **c**). The black line shows the size dependence of eutectic points.

Although the Kelvin effect is commonly considered for liquid-vapour equilibrium and phase partitioning, size effects on the liquid-solid equilibrium upon phase transitions have been rarely taken into account in atmospheric thermodynamic and kinetic models. On the basis of the above results, we propose to include the particle dry diameter (*D*_s_) as the third dimension in the liquid–solid equilibrium phase diagrams of aerosol particles. Figure 4a shows such a 3-D phase diagram for the AS-water system. In Fig. 4a, the surfaces represent the equilibrium between liquid and crystalline phases. It is estimated from polynomial fitting to literature data (solid circles). A slice on the surface at *D*_s_^−1^=0 (*D*_s_→∞) equals the traditional *T*-*x*_s_ diagram for bulk material as illustrated in Fig. 4b.

A slice on the surface at constant *T* provides a *D*_s_^−1^-*x*_s_ phase diagram showing the influence of particle size on equilibrium composition. As an example, Fig. 4c shows a *D*_s_^−1^-*x*_s_ slice at 215 K, which exhibits a similar pattern as the traditional *T*-*x*_s_ diagram in Fig. 4b. Figure 4d displays the *D*_s_^−1^-*x*_s_ diagram at 298 K, which contains the observed size-dependent solubility of AS particles reflecting the Ostwald–Freundlich effect^15^. Note that, at this temperature, the ice phase does not exist. Extrapolation of the experimental data for aqueous AS to *x*_s_=1 leads to a critical melting diameter of ~4 nm, that is, pure AS particles below this size are expected to be in a liquid state at 298 K.

Finally, a slice at constant *x*_s_ gives a *T*-*D*_s_^−1^ phase diagram showing the effect of particle size on phase transition temperature (Fig. 4e). For example, in a system with an AS mass fraction of *x*_s_=0.63, the characteristic temperature for complete dissolution decreases from ~470 K for bulk material to ~298 K for 10-nm particles. At *x*_s_=0 or 1, the diagram becomes a *T*-*D*_s_^−1^ diagram for a pure substance, reflecting the Gibbs–Thomson effect^20^. Related effects of particle size on melting, freezing and glass transitions have been reported and quantified for a wide range of materials such as water, organic compounds and metals^21^,^22^,^23^ (Supplementary Fig. 4). Our results demonstrate that similar effects and formalisms also apply to other phase transition processes and mixed systems, such as liquid–solid phase separation in salt-water droplets. The size-dependent phase transition temperature and increased solubility of nanoparticles reflect a tendency of smaller particles for staying in liquid and mixed phase compared with bulk materials (Supplementary Fig. 5). This may partly explain the unresolved size-dependent morphology of organic-AS particles, that is, smaller particles are internally mixed while larger particles adopt a de-mixed partially engulfed structure^10^.

## Discussion

**Figure 5:**
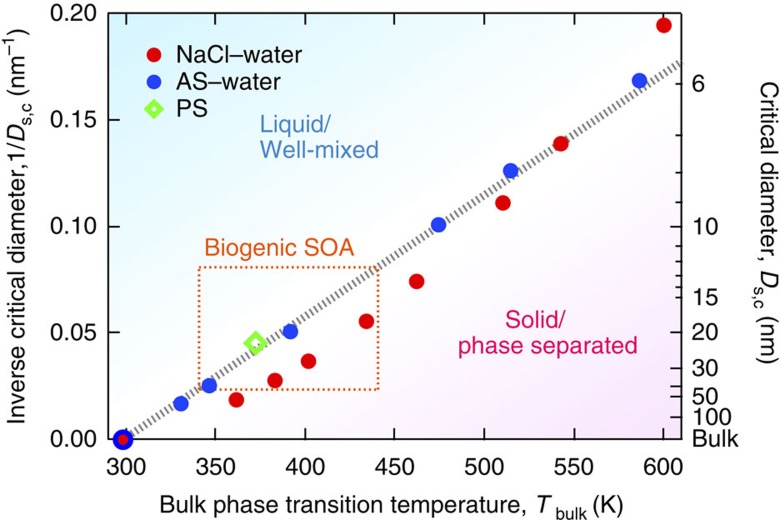
Dependence of critical diameter on bulk phase transition temperature. Inverse critical diameters of liquefaction at 298 K (*D*_s,c_^−1^) are plotted against bulk phase transition temperatures (*T*_bulk_) for aqueous ammonium sulphate (AS, blue solid circle), aqueous sodium chloride (NaCl, red solid circle) and low chain length polystyrene (PS, green open diamond)^22^. The data points are observations and the dotted line is a linear fit to all data through the point of [298 K, *D*_s_^−1^=0]. The orange dashed line bounded area indicates the parameter range estimated for atmospheric biogenic secondary organic aerosol (SOA).

According to the size effects outlined above, aerosol particles of identical chemical composition may coexist in different states in the atmosphere depending on particle diameter. Thus, we are interested in the critical diameter (*D*_s,c_) below which aerosol particles are expected to be liquid and well-mixed at ambient temperature. Using *T*-*D*_s_^−1^ diagrams like Fig. 4e for particles with different chemical composition (Supplementary Fig. 6), we obtained critical dry diameters as a function of bulk phase transition temperature (*T*_bulk_). As shown in Fig. 5, all data for aqueous AS and NaCl particles available at 298 K converge onto a compact near-linear correlation of 1/*D*_s,c_ versus *T*_bulk_. The glass transition of low chain length polystyrene^22^ as the only organic reference material (with low molecular weight) for which similar substrate-free data are currently available falls onto the same correlation line. These findings suggest that the influence of particle size on the liquefaction of substances is related to bulk phase transition temperature in a similar way for different salts as well as organic compounds, implying a close relationship between interfacial energy and the enthalpy of phase transition similar to the Turnbull relation^24^ (see Supplementary Fig. 7 and Supplementary Discussion for more details).

Chamber experiments with pine emissions-derived secondary organic aerosol (SOA) as well as field measurements of atmospheric biogenic SOA indicate a change of particle phase state at ~20–30 nm (refs 3, 25), suggesting that particles below this limit are in the liquid-like state. This observation appears consistent with a critical diameter around ~20 nm (12–40 nm), which can be derived from the estimated bulk melting temperatures of individual functionalized acids found in biogenic SOA (~340–440 K, orange dashed line bounded area in Fig. 5, see Supplementary Note 4)^2^. This consistency increases confidence in the similarity of *T*_bulk_-*D*_s,c_ relations. SOA particles usually consist of mixtures of multiple compounds, which reduces the bulk melting temperature^26^, thus increasing the critical diameter and implying that SOA particles in the nucleation mode are very likely always liquid (of potentially high viscosity). The liquid-to-solid transition of SOA particles observed at ~20–30 nm was suggested to be a glass transition^3^,^25^, for which the transition temperature is usually by a factor of ~0.7 lower than for melting^2^. Explaining the observed transition would thus require a fairly high bulk glass transition temperature (~370 K), suggesting that the SOA particles may contain oligomers or polymers, in agreement with independent observations^27^.

The methods and results presented in this study advance the understanding and challenge the traditional modelling of atmospheric aerosol phase state and processing. The size effects on the phase states may have important implications for a number of atmospheric interfacial and condensed phase processes such as nucleation, water/gas uptake, polymerization and heterogeneous reactions^2^,^27^,^28^,^29^. The combination of the DKA method and tandem differential mobility analyser technique can be used to study the thermodynamic properties of nanoparticles from a wide range of materials and enables substrate-free experimental investigation of nanoparticles not only on humidification and drying but also with regard to other types of conditioning, for example, exposure to organic solvents, heat treatment and volatilization^30^,^31^. Thus, the scientific approach presented here may be useful for nanoparticle studies in various fields, including the environmental, biomedical and materials sciences^20^,^32^.

## Methods

### HTDMA experiments

Our analysis is based on the Harvard hygroscopic tandem differential mobility analyser (HTDMA) measurements^8^,^9^, where a mono-disperse dry particle fraction of dry diameter *D*_s_ is selected by the first DMA, equilibrated to a defined relative humidity (equivalent to water vapour saturation ratio *s*_w_=RH/100%) and finally goes through a second DMA for the determination of the humidified size distribution. The HTDMA results were usually reported in the form of the growth factor *g*_f_ as a function of *s*_w_ at each *D*_s_. Details of shape factor correction of HTDMA data can be found in Supplementary Note 1. Data from the efflorescence mode experiments (containing the most concentrated droplet solutions) are adopted for the DKA analysis.

### Differential Köhler analysis (DKA)

The theoretical basis of the DKA method is the Köhler equation^33^, which describes the equilibrium *s*_w_ over a spherical droplet as a function of *a*_*w*_, *σ*_lv_ and the droplet size *D*_sol_


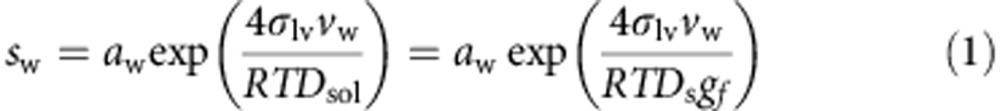


where *v*_w_ is the partial molar volume of water (Supplementary Note 1), *R* is the universal gas constant and *T* is temperature. *D*_sol_ in equation (1) is often replaced by the product of *D*_s_ and *g*_f_, in which *D*_s_ is the dry diameter of spherical solute particles and *g*_f_ is the particle geometrical diameter growth factor, that is, *g*_f_=*D*_sol_/*D*_s_. As parameters *s*_w_, *D*_s_ and *g*_f_ can be determined from the HTDMA measurements, *σ*_lv_ and *a*_w_ (considered as size-independent) become the only unknowns in equation (1). Note that *σ*_lv_ and *a*_w_ are both functions of *g*_f_ (*g*_*f*_ represents the solute concentration *x*_s_, see Supplementary Note 1). For each *x*_s_ (or *g*_*f*_), we need to construct at least two independent equations by performing HTDMA measurements at different *D*_s_ to realize the differential analysis as follows. Converting equation (1) into logarithmic form, we have


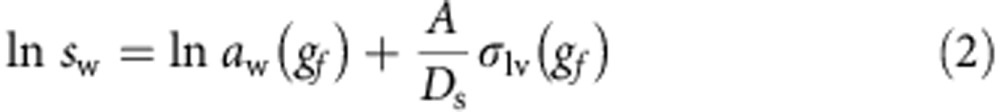


where *A*=4*v*_w_/(*RTg*_*f*_). With measurements at two dry diameters *D*_s1_ and *D*_s2_, we have


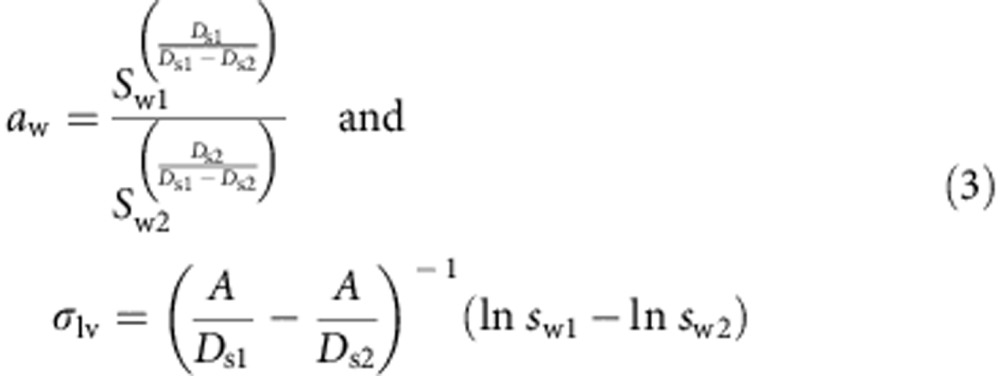


where *s*_w1_ and *s*_w2_ are measured at the same *g*_f_ but at *D*_s1_ and *D*_s2_, respectively. When measurements at the same *g*_f_ are available for more than two initial particle dry diameters, a multiple regression was applied to all sizes resulting in a best estimate of *a*_w_ and *σ*_lv_. More details about the DKA method can be found in Supplementary Note 1.

### Modelling of deliquescence and efflorescence

Deliquescence of salt particle of dry diameter *D*_s_ happens when the ambient *s*_w_ equals the equilibrium *s*_w_ over a droplet of the wet diameter equal to *D*_s_, and saturated with respect to crystalline salt particles of size *D*_s_. The deliquescence concentration b* was determined by the Ostwald–Freudlich equation^15^ through corresponding deliquescence solute activity *a*_s_*


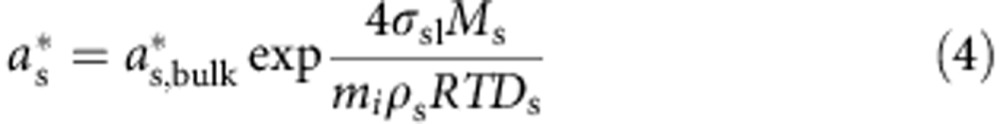


in which *a*_s_* is the solute activity in a solution saturated with respect to salt particles of diameter *D*_s_, *a*_s,bulk_* is the solute activity in saturated bulk solution, *M*_s_ is molar weight of solute, *ρ*_s_ is the density of solute and *m*_i_ is the stoichiometric number of dissociated ions. The *σ*_sl_ is determined to be 0.156 and 0.265 N m^−1^ for AS and NaCl, respectively (Supplementary Note 2). Substituting b* into the Köhler equation (1) gives the DRH.

The efflorescence is predicted based on classical nucleation theory^16^,^17^,^34^ along with the Köhler equation^33^. Given a Köhler curve, the volume of the droplet *V*_sol_ and corresponding *b* at each point can be easily determined. Then we can calculate the nucleation rate *ω* at each point by^16^


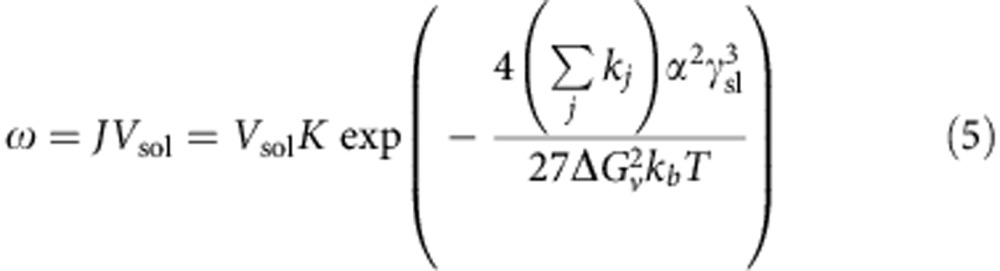


where *V*_sol_ is the volume of the droplet, *K* is the pre-exponential factor, *k*_*j*_ and *α* are geometrical constants dependent on the morphology of the particular crystal, Δ*G*_v_ is the excess free energy of solute per unit volume in the crystalline phase over that in solution, *k*_b_ is the Boltzmann constant. We assumed that the formation of a single critical-sized nucleus is sufficient to initiate the crystallization of the entire droplet and trigger the efflorescence. Then ERH and *b*_e_ can be determined from the point where *ωt*_i_=1 (*t*_i_=2 s is the induction time interval for the investigated system^9^) (Fig. 3). We performed model predictions of DRH and ERH over the entire size range of 6–60 nm for AS and NaCl by this approach with *σ*_*s*l_ and *γ*_*s*l_ determined at one particular diameter (6 nm, Supplementary Note 2).

## Author contributions

Y.C. and H.S. designed and performed the study. T.K., E.M. and U.P. discussed the results, interpretation and implications and commented on the manuscript at all stages. Y.C. and H.S. wrote the supplement. Y.C., H.S. and U.P. wrote the manuscript with inputs from all the co-authors.

## Additional information

**How to cite this article**: Cheng, Y. *et al.* Size dependence of phase transitions in aerosol nanoparticles. *Nat. Commun.* 6:5923 doi: 10.1038/ncomms6923 (2015).

## Supplementary Material

Supplementary Information Supplementary Figures 1-7, Supplementary Table 1, Supplementary Notes 1-4, Supplementary Discussion and Supplementary References

## References

[b1] Martin S. T. Phase transitions of aqueous atmospheric particles. Chem. Rev. 100, 3403–3454 (2000). 11777428 10.1021/cr990034t

[b2] Koop T., Bookhold J., Shiraiwa M. & Poschl U. Glass transition and phase state of organic compounds: dependency on molecular properties and implications for secondary organic aerosols in the atmosphere. Phys. Chem. Chem. Phys. 13, 19238–19255 (2011). 21993380 10.1039/c1cp22617g

[b3] Virtanen A. *et al.* An amorphous solid state of biogenic secondary organic aerosol particles. Nature 467, 824–827 (2010). 20944744 10.1038/nature09455

[b4] Pöschl U. Atmospheric aerosols: composition, transformation, climate and health effects. Angew. Chem. Int. Ed. 44, 7520–7540 (2005). 10.1002/anie.200501122 16302183

[b5] Krieger U. K., Marcolli C. & Reid J. P. Exploring the complexity of aerosol particle properties and processes using single particle techniques. Chem. Soc. Rev. 41, 6631–6662 (2012). 22739756 10.1039/c2cs35082c

[b6] Russell L. M. & Ming Y. Deliquescence of small particles. J. Chem. Phys. 116, 311–321 (2002).

[b7] Orr C. Jr, Hurd F. K. & Corbett W. J. Aerosol size and relative humidity. J. Colloid Sci. 13, 472–482 (1958).

[b8] Biskos G., Russell L. M., Buseck P. R. & Martin S. T. Nanosize effect on the hygroscopic growth factor of aerosol particles. Geophys. Res. Lett. 33, L07801 (2006).

[b9] Biskos G., Paulsen D., Russell L. M., Buseck P. R. & Martin S. T. Prompt deliquescence and efflorescence of aerosol nanoparticles. Atmos. Chem. Phys. 6, 4633–4642 (2006).

[b10] Veghte D. P., Altaf M. B. & Freedman M. A. Size dependence of the structure of organic aerosol. J. Am. Chem. Soc. 135, 16046–16049 (2013). 24125549 10.1021/ja408903g

[b11] Damson A. W. & Gast A. P. Physical Chemistry of Surfaces 6th edn John Wiley & Sons, Inc. (1997).

[b12] Vicente C., Yao W., Maris H. J. & Seidel G. M. Surface tension of liquid ^4^He as measured using the vibration modes of a levitated drop. Phys. Rev. B 66, 214504 (2002).

[b13] Tang I. N., Munkelwitz H. R. & Wang N. Water activity measurements with single suspended droplets: The NaCl-H_2_O and KCl-H_2_O systems. J. Colloid Interf. Sci. 114, 409–415 (1986).

[b14] Chan C. K., Liang Z., Zheng J., Clegg S. L. & Brimblecombe P. Thermodynamic properties of aqueous aerosols to high supersaturation: I-measurements of water activity of the system Na^+^−Cl^−^−NO_3_^−^−SO_4_^2-^−H_2_O at~298.15K. Aerosol Sci. Tech. 27, 324–344 (1997).

[b15] Ostwald W. Über die vermeintliche isomerie des roten und gelben Quecksilbersoxyds und die Oberflächenspannung fester Körper. Z. Phys. Chem. 34, 495–503 (1900).

[b16] Cohen M. D., Flagan R. C. & Seinfeld J. H. Studies of concentrated electrolyte solutions using the electrodynamic balance. 3. Solute nucleation. J. Phys. Chem. 91, 4583–4590 (1987).

[b17] Gao Y., Chen S. B. & Yu L. E. Efflorescence relative humidity of airborne sodium chloride particles: A theoretical investigation. Atmos. Environ. 41, 2019–2023 (2007).

[b18] Koop T., Luo B., Tsias A. & Peter T. Water activity as the determinant for homogeneous ice nucleation in aqueous solutions. Nature 406, 611–614 (2000). 10949298 10.1038/35020537

[b19] Dutcher C. S., Wexler A. S. & Clegg S. L. Surface tensions of inorganic multicomponent aqueous electrolyte solutions and melts. J. Phys. Chem. A 114, 12216–12230 (2010). 21043484 10.1021/jp105191z

[b20] Alcoutlabi M. & McKenna G. B. Effects of confinement on material behaviour at the nanometre size scale.. J. Phys.: Condens. Matter 17, R461 (2005).

[b21] Couchman P. R. & Jesser W. A. Thermodynamic theory of size dependence of melting temperature in metals. Nature 269, 481–483 (1977).

[b22] Forrest J. A. & Mattsson J. Reductions of the glass transition temperature in thin polymer films: Probing the length scale of cooperative dynamics. Phys. Rev. E 61, R53–R56 (2000). 10.1103/physreve.61.r53 11046371

[b23] Pan D., Liu L.-M., Slater B., Michaelides A. & Wang E. Melting the ice: on the relation between melting temperature and size for nanoscale ice crystals. ACS Nano 5, 4562–4569 (2011). 21568289 10.1021/nn200252w

[b24] Turnbull D. Formation of crystal nuclei in liquid metals. J. Appl. Phys. 21, 1022–1028 (1950).

[b25] Virtanen A. *et al.* Bounce behavior of freshly nucleated biogenic secondary organic aerosol particles. Atmos. Chem. Phys. 11, 8759–8766 (2011).

[b26] Marcolli C., Luo B. & Peter T. Mixing of the organic aerosol fractions: liquids as the thermodynamically stable phases. J. Phys. Chem. A 108, 2216–2224 (2004).

[b27] Kalberer M. *et al.* Identification of polymers as major components of atmospheric organic aerosols. Science 303, 1659–1662 (2004). 15016998 10.1126/science.1092185

[b28] Murray B. J. *et al.* Heterogeneous nucleation of ice particles on glassy aerosols under cirrus conditions. Nat. Geosci. 3, 233–237 (2010).

[b29] Shiraiwa M., Ammann M., Koop T. & Poschl U. Gas uptake and chemical aging of semisolid organic aerosol particles. Proc. Natl Acad. Sci. USA 108, 11003–11008 (2011). 21690350 10.1073/pnas.1103045108 PMC3131339

[b30] Joutsensaari J., Vaattovaara P., Vesterinen M., Hämeri K. & Laaksonen A. A novel tandem differential mobility analyzer with organic vapor treatment of aerosol particles. Atmos. Chem. Phys. 1, 51–60 (2001).

[b31] Cheng Y. F. *et al.* Size-resolved measurement of the mixing state of soot in the megacity Beijing, China: diurnal cycle, aging and parameterization. Atmos. Chem. Phys. 12, 4477–4491 (2012).

[b32] Rabinow B. E. Nanosuspensions in drug delivery. Nat. Rev. Drug Discov. 3, 785–796 (2004). 15340388 10.1038/nrd1494

[b33] Köhler H. The nucleus in and the growth of hygroscopic droplets. Trans. Faraday Soc. 32, 1152–1161 (1936).

[b34] Gao Y., Chen S. B. & Yu L. E. Efflorescence relative humidity for ammonium sulfate particles. J. Phys. Chem. A 110, 7602–7608 (2006). 16774203 10.1021/jp057574g

[b35] Tang I. N. & Munkelwitz H. R. Water activities, densities, and refractive indices of aqueous sulfates and sodium nitrate droplets of atmospheric importance. J. Geophys. Res-Atmos. 99, 18801–18808 (1994).

[b36] Tang I. N. Chemical and size effects of hygroscopic aerosols on light scattering coefficients. J. Geophys. Res-Atmos. 101, 19245–19250 (1996).

[b37] Seinfeld J. H. & Pandis S. N. Atmospheric Chemistry and Physics, from Air Pollution to Climate Change John Wiley (2006).

[b38] Clegg S. L., Brimblecombe P. & Wexler A. S. Thermodynamic Model of the System H^+^−NH_4_^+^−Na^+^−SO_4_^2-^−NO_3_^-^−Cl^-^−H_2_O at 298.15K. J. Phys. Chem. A 102, 2155–2171 (1998).

[b39] Pruppacher H. R. & Klett J. D. Microphysics of clouds and precipitation Kluwer Academic Publishers (1997).

[b40] Clegg S. L., Ho S. S., Chan C. K. & Brimblecombe P. Thermodynamic properties of aqueous (NH_4_)_2_SO_4_ to high supersaturation as a function of temperature. J. Chem. Eng. Data 40, 1079–1090 (1995).

[b41] Kreidenweis S. M. *et al.* Water activity and activation diameters from hygroscopicity data-Part I: Theory and application to inorganic salts. Atmos. Chem. Phys. 5, 1357–1370 (2005).

[b42] Xu J., Imre D., McGraw R. & Tang I. Ammonium sulfate: equilibrium and metastability phase diagrams from 40 to −50°C. J. Phys. Chem. B 102, 7462–7469 (1998).

[b43] Kendall J. & Davidson A. W. The melting point of ammonium sulfate. J. Ind. Eng. Chem. 13, 303–304 (1921).

