# Supplementary material for: Size dependence of phase transitions in aerosol nanoparticles
Source: Nat Commun. 2015 Jan 14;6:5923. doi: 10.1038/ncomms6923 (PMC4309446; doi:10.1038/ncomms6923)
Supplement: Supplementary Information — Supplementary Figures 1-7, Supplementary Table 1, Supplementary Notes 1-4, Supplementary Discussion and Supplementary References [file ncomms6923-s1.pdf]

Supplementary Figures

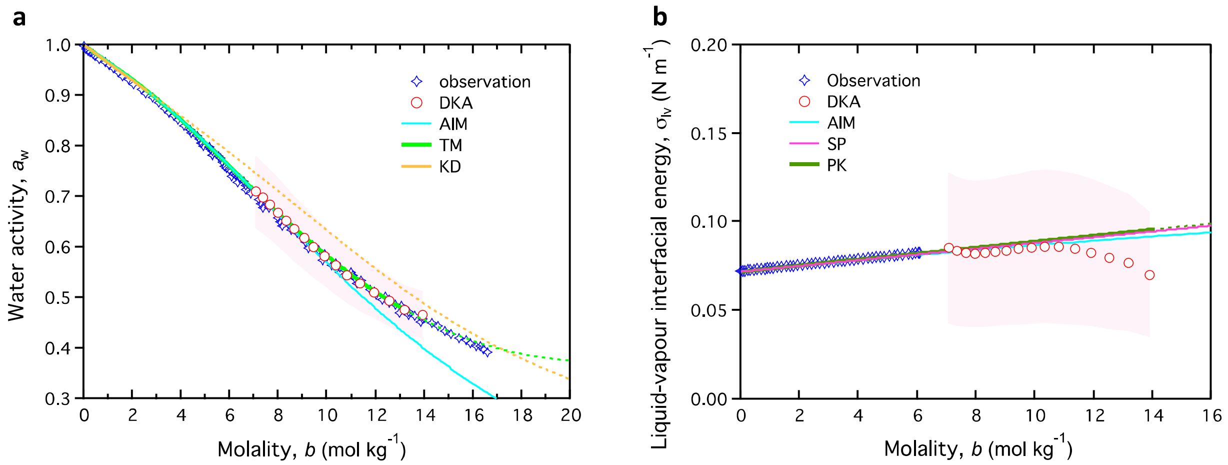

Supplementary Figure 1 | Concentration-dependent water activity and liquid-vapour interfacial energy of aqueous sodium chloride solution. a, b, Water activity (a_w) and liquid-vapour interfacial energy (σ_{lv}) are plotted against solute molality (b). The DKA-derived a_w and σ_{lv} (red open circles) are compared with observations (blue stars)[1-5], Aerosol Inorganic Model[6] (AIM, blue line), and other parameterisations for a_w (TM = Tang-Munkelwitz[7], green line; KD = Kreidenweis[8], orange line) and σ_{lv} (PK = Pruppacher-Klett[5], dark green line; SP = Seinfeld-Pandis[9], purple line) (Supplementary Note 3). Dashed lines indicate extrapolation beyond validated concentration range. Pink shaded areas indicate the uncertainties in the DKA retrieval, estimated by Monte Carlo analyses (Supplementary Note 1).

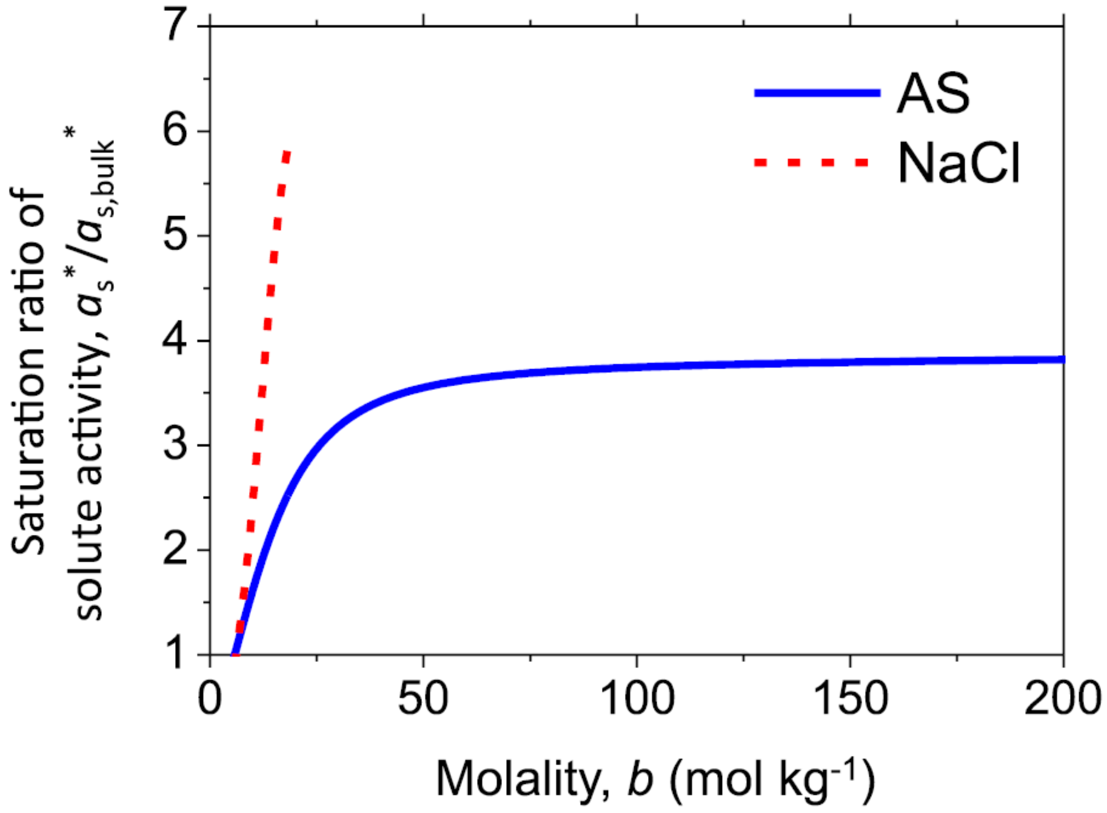

Supplementary Figure 2 | Saturation ratio of solute activity ($a_s^*/a_{s,bulk}^*$) as a function of molality b for ammonium sulphate (AS) and sodium chloride (NaCl).

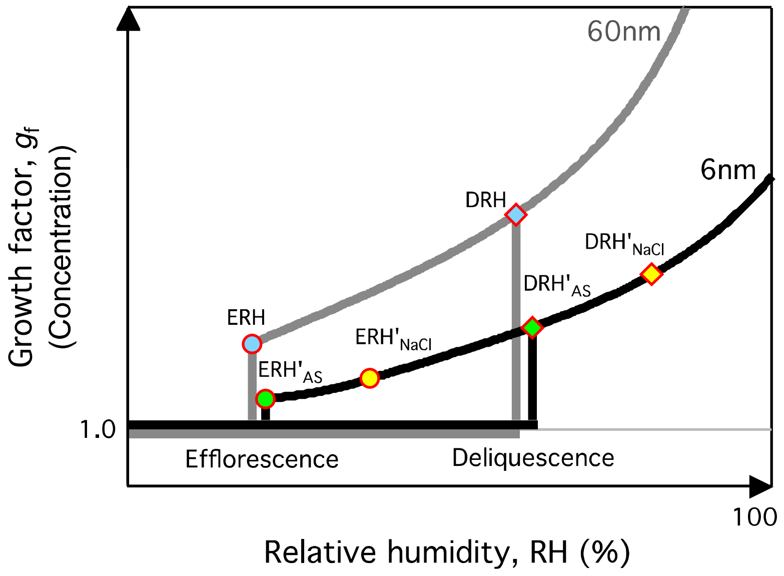

Supplementary Figure 3 | Schematic phase diagram of nanoparticles, exampled for 6-nm and 60-nm. According to Köhler theory, smaller growth is expected for smaller particles upon humidification due to the curvature Kelvin effect. Theoretically, efflorescence of a supersaturated droplet happens when its concentration reaches its characteristic crystallization concentration (molality b_e). The efflorescence relative humidity (ERH) can be determined from b_e along the Köhler curve. If the b_e values (equivalent to the growth factor g_f upon efflorescence, see Supplementary Note 1) are the same for particles of different sizes (size-independent), the ERH will be size-dependent because the Köhler curves do not overlap each other, and vice versa, i.e., if ERH is not size-dependent, b_e and g_f upon efflorescence must be size-dependent. Analogously, similar facts hold for the deliquescence concentration and relative humidity (DRH). Specifically, the phase transition concentrations of ammonium sulphate (AS) increase tremendously when the particle diameter decreases from 60-nm to 6-nm, leading to the almost unchanged DRH and ERH. While for sodium chloride (NaCl), the size dependence of its phase transition concentrations is much weaker than that of AS, resulting in more prominent shift in its ERH and DRH.

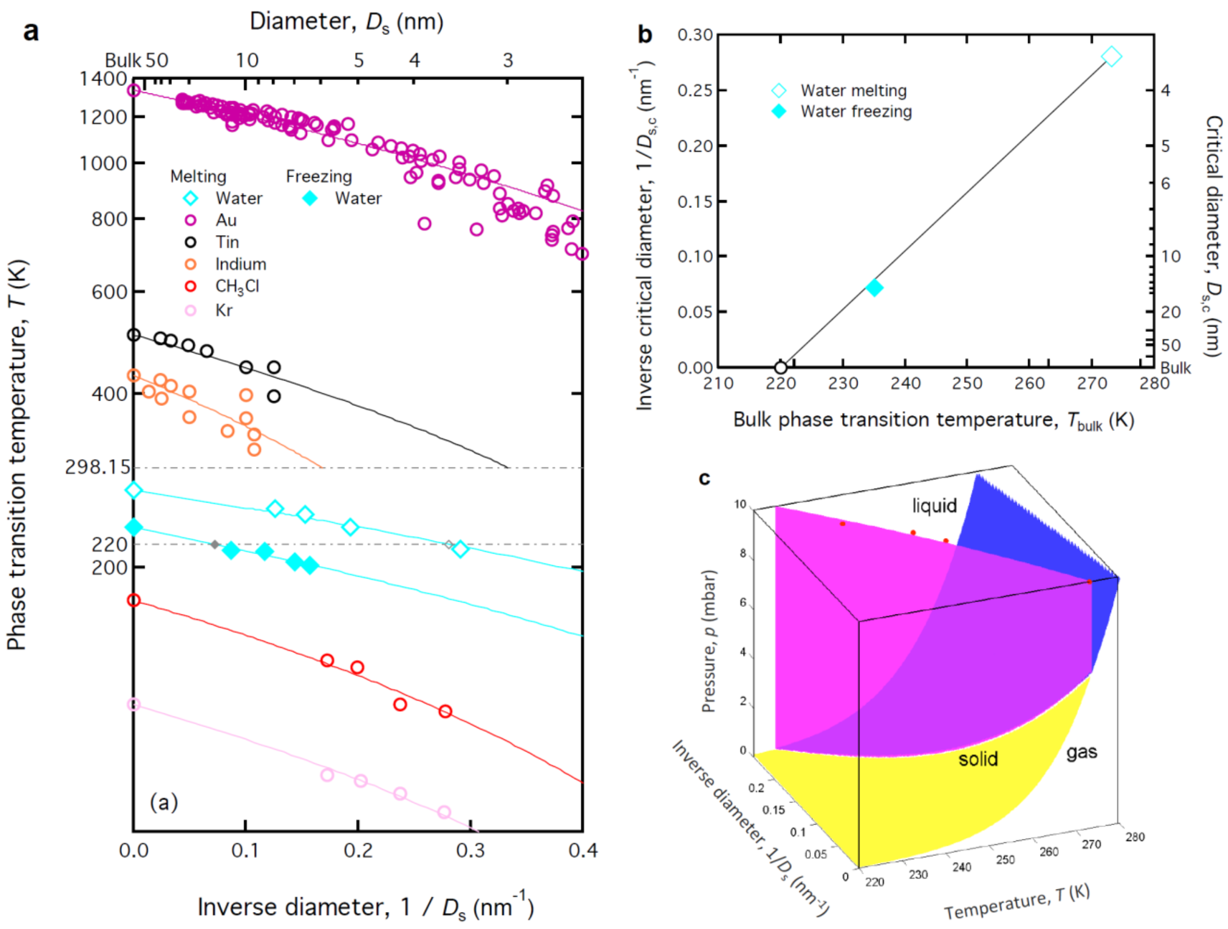

Supplementary Figure 4 | Size effect on various phase transition processes. a, Size-dependent phase transition temperature, such as melting temperature of metals (e.g., Au, Tin and Indium), water, methyl chloride CH_3Cl and Kr, as well as freezing temperature of water[10-16]. **b,** Similar to Fig. 5 but for water at 220 K, corresponding to solid and open grey diamonds on the grey dash line at 220 K in (**a**). The blue solid diamond represents the critical diameter ($D_{s,c}$), which can reduce the freezing temperature of water from 230 K (bulk) to 220 K. The blue open diamond represents the critical diameter, which can reduce the melting temperature of water from 273 K (bulk) to 220 K. They show a rather compact near-linear relationship with the starting point [220 K, D_s^{-1}=0] (black open circle). **c,** 3-D phase diagram for pure water in the coordinates of pressure (p), temperature (T) and inverse diameter (D_s^{-1}). The red solid circles represent the size-dependent melting temperature of water[11]. To facilitate plotting, it is assumed that the size dependence of water melting temperature is independent from pressure change in the most relevant pressure range.

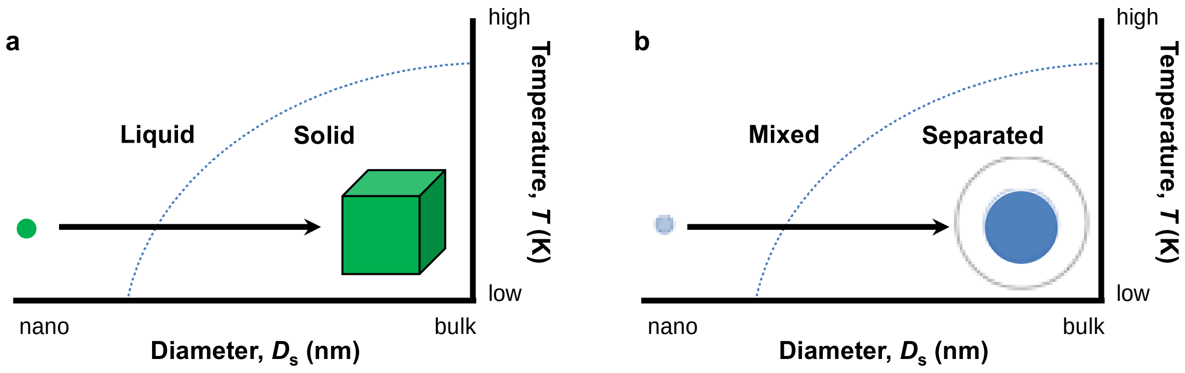

Supplementary Figure 5 | Illustration of size effects on the phase state of pure material (a) and mixed system (b). The dash lines represent the temperature of phase transition/separation as a function of particle size.

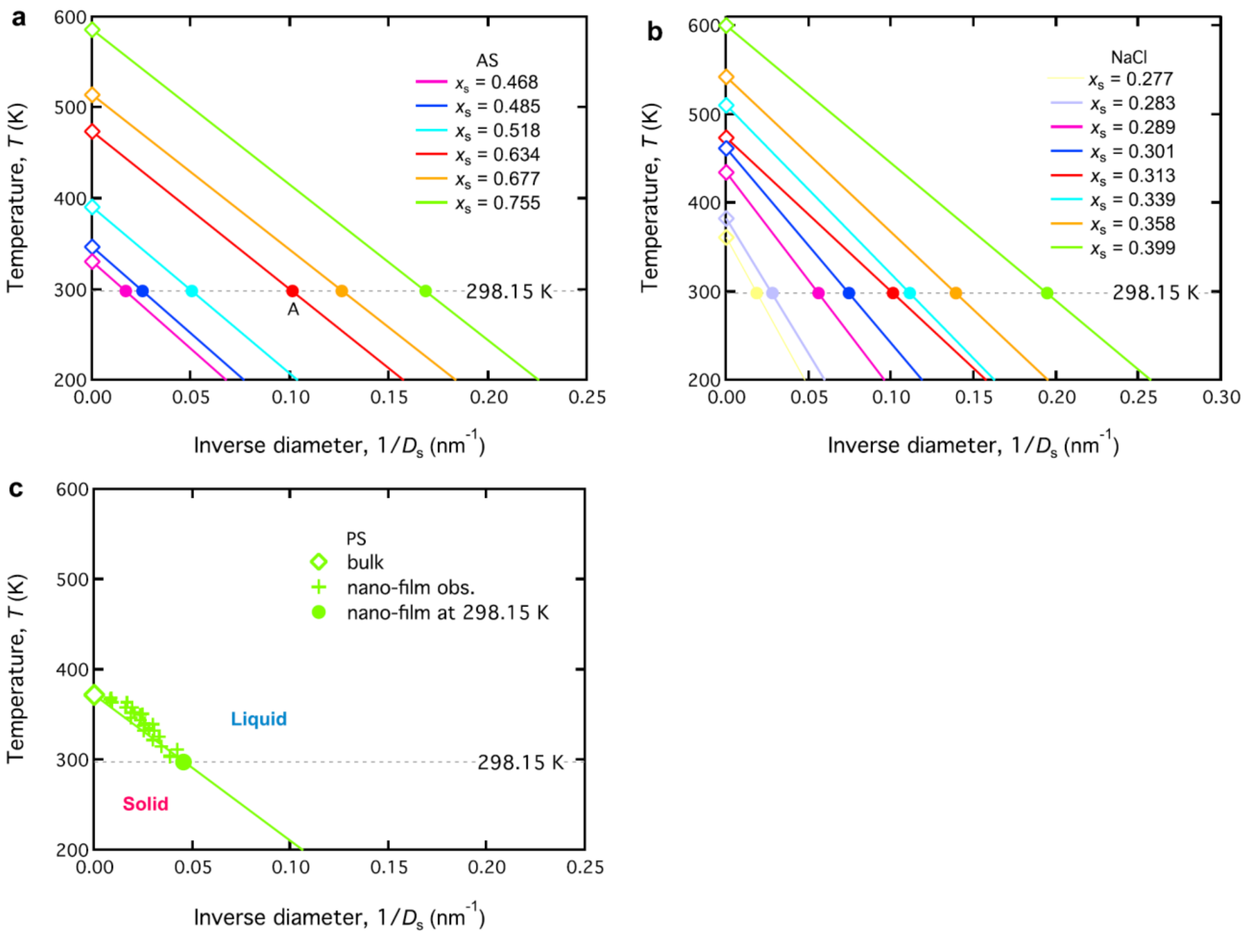

Supplementary Figure 6 | Liquid-solid equilibrium phase diagrams in the coordinate of inverse diameter ($1/D_s$) and phase transition temperature (T) of aqueous ammonium sulphate (AS), aqueous sodium chloride (NaCl) and low chain length polystyrene (PS). a, Slices at different AS mass fractions (x_s) from the 3-D phase diagram of AS-water system (Fig. 4a), similar to the red line in Fig.4e. The x_s values are selected according to the observed solubility of nanoparticles at different sizes. **b,** Similar plot for NaCl-water system. **c,** Size-dependent glass transition temperature of low chain length PS[17].

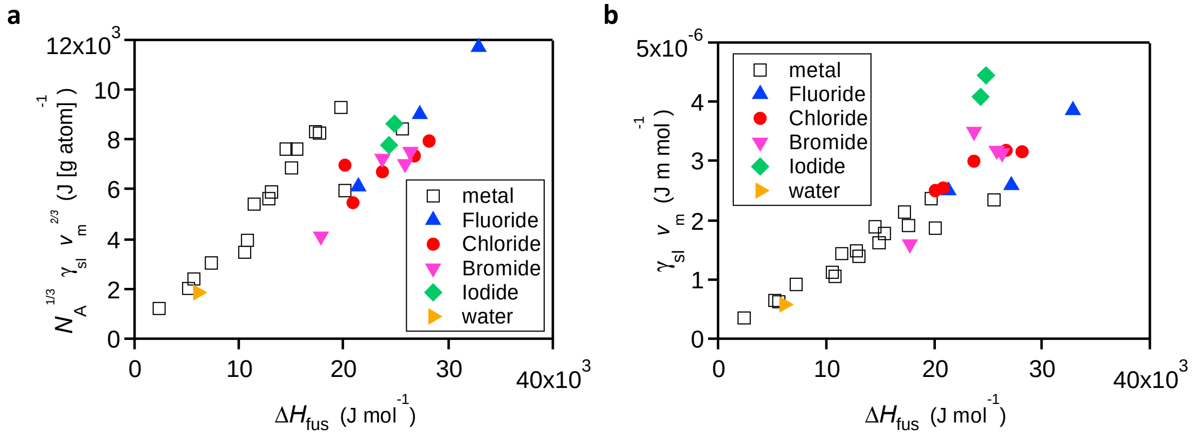

Supplementary Figure 7 | Comparison of enthalpy of fusion (ΔH_{fus}) with interfacial energy of embryo (γ_{sl}). ΔH_{fus} is compared to (**a**) $N_A^{1/3}\gamma_{sl}V_m^{2/3}$ as in Turnbull[18] and (**b**) $\gamma_{sl}V_m$. N_A denotes Avogadro constant, V_m is the molar volume. The metal and water data are taken from Turnbull[18] while the rest are taken from Buckle and Ubbelohde[19,20]. The metals include gold, silver, mercury, lead, etc. The chloride, fluoride, bromide and iodide correspond to LiCl, NaCl, KCl, RbCl, CsCl; LiF, NaF, CsF; LiBr, NaBr, Kbr, CsBr; and KI, CsI, respectively.

Supplementary Tables

Supplementary Table 1 | Fit equations for the DKA-retrieved water activity a_w and liquid-vapour interfacial energy σ_{lv} (N m^{-1}) of ammonium sulphate (AS) at 298K. Here, b is the molality (mol kg^{-1}) and x_s is the AS mass fraction.

Equations	Parameters
$a_w = \frac{A_0 + A_1 x + A_2 x^2}{B_0 + B_1 x + B_2 x^2 + x^3}$ and $x = \frac{b - 13.02}{17.15}$	A_0=46.29; A_1=26.42; A_2=12.51;<br>B_0= 80.77; B_1= 103.6; B_2= 55.09
$\sigma_{lv} = 0.072 + \sum C_i {x_s}^i$	C_1= 0.04070; C_2= -0.4627; C_3= 3.799;<br>C_4= -14.46; C_5= 27.80; C_6= -24.63; C_7= 8.016

Supplementary Note 1. Differential Köhler Analysis (DKA)

Shape factor correction for HTDMA measurement data. The results of humidified tandem differential mobility analyzer (HTDMA) were usually reported in the form of the growth factor g_f as a function of water vapour saturation ratio s_w at each dry equivalent spherical mobility diameter D_s. However, g_f and D_s determined by DMA need to be corrected for the particle shape (non-spherical) and porosity. For ammonium sulphate (AS), we adopted the method of Biskos et al.[21], taking a shape factor of 1.02 for the dry AS particle sizes and 1.0 for the wet particle sizes (no correction). For sodium chloride (NaCl) dry particles, we adopted a size-dependent shape factor varying from 1.24 to 1.07 as suggested by DeCarlo et al.[22] and Biskos et al.[23] for 8-nm to 60-nm NaCl nanoparticles. For 6-nm NaCl particles, the same correction method failed to reproduce the observed g_f over the whole hygroscopic growth curve. Biskos et al.[23] suggested that it is due to nanosize effects on thermodynamic properties and/or uncertainties in thermodynamic properties/shape factors. We notice that the deviation still exists for the largest droplet (e.g., with g_f = 1.6 measured at 6-nm and thus $D_{sol} = 6\times1.6 = 9.6$ nm), where the size is beyond the range of significant nanosize effects (on thermodynamic properties). It is likely that the shape factor is the main reason for the deviation.

In the present study, a shape factor of ~1.37 was applied to 6-nm NaCl particles. It was determined by numerically searching for a value that is able to match the observed hygroscopic growth curve with relative humidity (RH) higher than the deliquescence point, an equivalent spherical mobility diameter about 5.14 nm is obtained for the dry particle after this shape factor correction. Considering the measurement uncertainty in RH and g_f, the derived shape factor for 6-nm NaCl particle may vary from 1.28 to 1.45. This is slightly larger than the numerically calculated dynamic shape factor of ideal cubic NaCl particles (~1.24) in the free-molecular flow regime[22,24], which may due to the imperfect nucleation and coagulation[25].

Size dependence of σ_{lv}. It is known that the liquid-vapour interfacial energy (σ_{lv}) depends not only on the solute concentration (mass fraction x_s) but also on the particle size[26],

$\sigma_{lv} = \varepsilon\ \sigma_{lv,bulk}$ (1)

where $\sigma_{lv,bulk}$ denotes the bulk interfacial energy, and ε denotes the size-dependent curvature adjustment factor. The size-dependence of σ_{lv} will introduce another term $\frac{2v_w}{RT}\frac{d\sigma_{lv}}{dD_{sol}}$ into the Köhler equation. The following deduction will show the origin of this size dependent term.

The change of Gibbs free energy (G) for the creation of a spherical droplet is

$$\Delta G = (\mu_l - \mu_v)\frac{V_w}{v_w} + \sigma_{lv} A_w \tag{2}$$

where μ_l and μ_v represent the chemical potentials of the liquid and its vapor, respectively, σ_{lv} is the liquid-vapour interfacial energy, and A_w is the surface area of the droplet, V_w is the droplet volume, and v_w is the molar volume of the liquid.

The criterion of equilibrium for a system of prescribed temperature and pressure is that ΔG has reached its minimum, so

$$\frac{d\Delta G}{dD} = \frac{(\mu_l - \mu_v)}{v_w}\frac{dV_w}{dD} + \frac{d(\sigma_{lv} A_w)}{dD} = 0 \tag{3}$$

where D is the droplet diameter, then

$$\frac{(\mu_l - \mu_v)}{v_w}\frac{d(\frac{1}{6}\pi D^3)}{dD} + \frac{d(\sigma_{lv}\pi D^2)}{dD} = 0 \tag{4}$$

The difference in the chemical potential is a function of vapor pressure p over the droplet and the equilibrium vapor pressure p^*, $(\mu_l - \mu_v) = -RT\ln(p/p^*)$, so supplementary equation (4) becomes

$$-\frac{RT\ln(\frac{p}{p^*})}{v_w}\frac{1}{2}\pi D^2 + (2\sigma_{lv}\pi D + \pi D^2\frac{d\sigma_{lv}}{dD}) = 0 \tag{5}$$

$$-RT\ln(\frac{p}{p^*})+\frac{4v_w\sigma_{\mathrm{lv}}}{D}+2v_w\frac{d\sigma_{\mathrm{lv}}}{dD}=0 \tag{6}$$

Finally, we have

$$s_{\mathrm{w}}=\exp(\frac{4\sigma_{\mathrm{lv}}v_w}{RTD}+\frac{2v_w}{RT}\frac{d\sigma_{\mathrm{lv}}}{dD}) \tag{7}$$

where $s_{\mathrm{w}} = p/p^*$, and σ_{lv} is a function of the droplet size D. By neglecting the size dependence of σ_{lv}, i.e., $d\sigma_{\mathrm{lv}}/dD$=0, supplementary equation (7) becomes the tradition Kelvin equation

$$s_{\mathrm{w}}=\exp(\frac{4\sigma_{\mathrm{lv}}v_w}{RTD}) \tag{8}$$

In our study, particle sizes are larger than 6 nm. The size dependent term $\frac{2v_w}{RT}\frac{d\sigma_{\mathrm{lv}}}{dD_{\mathrm{sol}}}$ is negligible (<1%) compared to the original term $4\sigma_{\mathrm{lv}}v_{\mathrm{w}}/(RTD_{\mathrm{sol}})$ for the investigated size ranges and is therefore not considered. Substituting supplementary equation (1) into equation (1), the solution in equation (3) becomes

$$a_w=\frac{s_{\mathrm{w}1}^{(\frac{D_{s1}\varepsilon_2}{D_{s1}\varepsilon_2-D_{s2}\varepsilon_1})}}{s_{\mathrm{w}2}^{(\frac{D_{s2}\varepsilon_1}{D_{s1}\varepsilon_2-D_{s2}\varepsilon_1})}} \quad \text{and} \quad \sigma_{lv,bulk}=\frac{D_{s1}D_{s2}\ln\frac{s_{\mathrm{w}1}}{s_{\mathrm{w}2}}}{A(D_{s2}\varepsilon_1-D_{s1}\varepsilon_2)} \tag{9}$$

where $A=4v_{\mathrm{w}}/(RTg_f)$, a_{w} is the water activity, $s_{\mathrm{w}1}$ and $s_{\mathrm{w}2}$ are water saturation ratios measured at the same g_f but at $D_{\mathrm{s}1}$ and $D_{\mathrm{s}2}$, respectively. In this study, ε is calculated according to Bahadur and Russell[26]. However, it is worth noticing that the size dependence of interfacial energy will not play a significant role unless the solution droplets are smaller than 5 to 6 nm[26].

Partial molar volume of water v_{w}. Partial molar volume of water v_{w} can be expressed as a function of solution density[27],

$$v_w=\frac{M_{\mathrm{w}}}{\rho_{\mathrm{sol}}}(1+\frac{d\ln\rho_{\mathrm{sol}}}{d\ln x_{\mathrm{s}}}) \tag{10}$$

where M_w is the molar mass of water, ρ_{sol} is the solution density and x_s is the solute mass fraction.

Conversion of growth factor g_f to solute mass fraction x_s. In this section we show that if the concentrations of droplet solutions are the same, their growth factors g_f would also be identical. The symbols are defined as follows: m_s and m_{sol} are the mass of solute and solution, V_s and V_{sol} are the volume of solute and solution, ρ_s and ρ_{sol} are the density of solute and solution and x_s is the mass fraction of solute, respectively. By definition, we have for spherical particles/droplets of homogeneous composition

$$g_f = (\frac{V_{sol}}{V_s})^{1/3} = (\frac{m_{sol}}{\rho_{sol} V_s})^{1/3} = (\frac{m_s}{x_s \rho_{sol} V_s})^{1/3} = (\frac{V_s \rho_s}{x_s \rho_{sol} V_s})^{1/3} = (\frac{\rho_s}{x_s \rho_{sol}})^{1/3} \tag{11}$$

Here, ρ_s is a constant and ρ_{sol} is a function of solution concentration x_s. Therefore, for a specific solute/solutes and with the assumption of a size-independent solution density of spherical particles, the same x_s in general means the same g_f and vice versa. Note that, nano-size effects on the density itself can result in a change in the density when the size gets really small. Such effects are, however, trivial (<1%) above a threshold diameter ~1 nm to 5 nm[23,28], and are therefore negligible for 6 nm to 60 nm particles investigated in the present study.

The smallest growth factor of 6-nm ammonium sulphate particle observed by the HTDMA experiments during dehydration prior to crystallization is about 1.04 (upon efflorescence of 6-nm ammonium sulphate particle)[21], which corresponds to a molality b of ~380 mol kg^{-1}. However, the retrieval using DKA is limited by the highest overlapped concentrations, corresponding to b ~160 mol kg^{-1} (x_s ~0.96).

Uncertainty analyses with Monte-Carlo simulation. To properly estimate the uncertainty of the proposed DKA method, we performed a Monte Carlo simulation by randomly varying all or a section of input parameters/data for the retrievals. Deviations for these input parameters/data were considered to be normally distributed around the original value with one relative standard deviation (*std*). A range of ±3*std* around the original value contains 99% of the values possible for the input parameter/data point according to the respective uncertainty due to assumptions in

the parameterization or arisen from the experiments, which represent a conservative estimate of the maximum uncertainties and ensure that the overall uncertainty estimation is not based on excessively large outliers. The uncertainties of the different input parameters and data are summarised here as: ±2% for relative humidity (RH), ±1% for dry diameter sizing (D_s) of AS, ±5% for D_s of NaCl due to shape factor uncertainties, ±1% for wet diameter sizing (D_{sol}), ±2.5% for growth factor (g_f) [21, 23].

40,000 runs of retrieval calculations were tested to be sufficient for convergence of the mean value and of the standard deviation. The discrepancy between the expected mean value of those 40,000 runs and the calculated value without any uncertainty variation of the inputs is less than 0.5%. Consequently, the retrieval calculations of σ_{lv} and a_w were made by randomly choosing 20,000 different sets of input parameters and data (such as diameter, growth factor, and relative humidity). Three times relative standard deviation (±3*std*) of the results from those 40,000 runs are considered as uncertainties (at 99% confidence level) for the output of the DKA method (i.e., a_w and σ_{lv}).

Supplementary Note 2. Determination of a_s, σ_{ls} and γ_{ls}

The solute activity a_s on a mole fraction basis is calculated by the Gibbs-Duhem equation[9,29,30] at constant temperature and pressure, where n_s and n_w represent the mole fraction of solute and water, respectively

$$n_s d\ln a_s + n_w d\ln a_w = 0 \tag{12}$$

Integrating supplementary equation (12) from $x_{s,bulk}^*$ to x_s

$$\ln\frac{a_s}{a_{s,bulk}*} = -\int_{x_{s,bulk}*}^{x_s}\frac{n_w}{n_s}d\ln a_w \tag{13}$$

where $x_{s,bulk}^*$ is the mass fraction of solute in a saturated bulk solution, and $a_{s,bulk}^*$ is the solute activity at $x_{s,bulk}^*$. Based on the retrieved a_w, we evaluated the integral in supplementary equation (13) numerically and extended it to the same concentration range as a_w.

The interfacial energy at the solid-liquid interface σ_{sl} is determined by the Ostwald-Freundlich equation [31,32] as

$$\sigma_{sl} = \frac{m_i \rho_s RTD_s}{4M_s}\ln\frac{a_s*}{a_{s,bulk}*} \tag{14}$$

where a_s^* is the solute activity in a solution saturated with respect to salt particles of diameter D_s, M_s is molar weight of solute, ρ_s is the density of solute and m_i is the stoichiometric number of dissociated ions. In this study, a_s^* is determined from deliquescence of salt nanoparticles by the Köhler equation and the Gibbs-Duhem equation. According to Chen[33], deliquescence of salt particle of D_s happens when the ambient s_w equals the equilibrium s_w over a droplet of the size D_s and saturated with respect to crystalline salt particles of size D_s. In practice, this equilibrium s_w is the onset deliquescence s_w (equivalent to deliquescence relative humidity, DRH) and can be determined by HTDMA measurements. Since s_w (=DRH) and D_{sol} (=D_s) are known, the equilibrium concentration b^* can be determined by equation (1) and b^* represents the saturation molality with respect to D_s. Then a_s^* and σ_{sl} can be determined. The size dependence of σ_{sl}

shows negligible impact on the simulation, i.e., <0.5% differences in the predicted μ_{del} and <0.2% differences in the DRH. It is hence neglected in our simulation.

The interfacial energy of solute embryo γ_{sl} is determined by the classical nucleation theory as Cohen et al.[29] and Gao et al.[34,35].

$$\gamma_{sl} = \left[\frac{4(\sum_j k_j)\alpha^2}{27\Delta G_v{}^2 k_b T}\ln(V_{sol}Kt_i)\right]^{-1/3} \tag{15}$$

where k_j and α are geometrical constants dependent on the morphology of the particular crystal, ΔG_v is the excess free energy of solute per unit volume in the crystalline phase over that in solution, k_b is the Boltzmann constant, V_{sol} is the volume of the droplet, K is the pre-exponential factor, and t_i is the induction time interval (taken as 2 second for the HTDMA measurements[21]). All parameters except a_w and a_s in supplementary equation (15) were taken the same as in Cohen et al.[29]. It is still on debate if a closure should be expected between σ_{sl} and γ_{sl}[25]. We found that $\sigma_{sl} > \gamma_{sl}$ for the investigated AS and NaCl salts particles which seems to hold for other compounds (e.g. $BaSO_4$) as well[36]. The fact that $\sigma_{sl} > \gamma_{sl}$ appears to be in line with the general size dependence of interfacial energies[26]: decreasing interfacial energy is expected for embryos of sizes ~1 nm[29] while no significant difference is expected for particles larger than 6 nm.

Supplementary Note 3. Thermodynamic models and parameterizations

Overview of model selection. In the following, we listed the reasons why the particular models were chosen for model prediction and comparison in our study:

(1) To represent the current study

We use newly determined a_w and σ_{lv} by the DKA method for AS. These data agree very well with literature observation data and also largely extend the concentration beyond the observation and existing parameterization methods as shown in Fig. 2.

For NaCl, we use a_w from the modified TM model (more details as given below) and σ_{lv} from the PK model[5]. This is because the DKA method suffers from uncertainties in the shape factor correction for NaCl particles, and the modified TM and PK agrees well with the DKA derived a_w and σ_{lv} for NaCl, as shown in Supplementary Fig. 1. The good agreement between modeled and observed deliquescence/efflorescence (Fig. 1) also confirms the reliability of modified TM and PK in the investigated concentration range.

The modified TM adopts the same expression as the original TM model[7] for the validated concentration range (x_s<0.45) and used an expression

$$a_w = \frac{0.1703 \cdot x_s^2 - 0.6018 \cdot x_s + 0.4315}{x_s^2 - 0.4594 \cdot x_s + 0.4343} \qquad (16)$$

for higher concentration range (x_s>0.45), which is constrained by available measurement data and the point [x_s=1, a_w=0]. The main reason for using supplementary equation (16) instead of the original TM is because the later model produces unrealistic results (a_w>1) at x_s>0.45.

(2) To represent Biskos et al.

We choose the original model used by Biskos et al.[21,23], i.e., a combination of TM and PK to represent their study. The molar volume of pure water was used instead of the partial molar volume as in Biskos et al.[21,23].

(3) AIM

The AIM growth curves in Fig. 1 are based on a_w and σ_{lv} from AIM (Aerosol Inorganics Model). There are two reasons for choosing AIM. First, AIM is one of the most commonly used and powerful models for studying the thermodynamic of inorganic aerosols. Second, AIM has the capacity to model highly supersaturated solution[37], providing a good reference under conditions where no measurement data are available. Such comparison could help to illustrate the performance of our method and its potential in improving the AIM model for highly supersaturated solution.

(4) Further comparison of different parameterization methods

Moreover, the TM and PK models are based on the best fit to the literature measurement data of a_w and σ_{lv} (Fig. 2 and Supplementary Fig. 1). As shown in Fig. 2a and Supplementary Fig. 1a, the agreement with measured a_w is better for the TM model than for the KD model[8] and AIM. The KD model is only used as a complete and proper reference to previous work.

The SP model[9] and the PK model were proposed to describe the concentration dependence of σ_{lv}. For NaCl, their difference is not significant within the concentration range of our simulation (Supplementary Fig. 1b). In Fig. 1, we showed only one simulation for clarity.

Aerosol Inorganics model (AIM). Aerosol Inorganics Model-III can be used to model the water activity a_w, liquid-vapour interfacial energy σ_{lv}, and density ρ_{sol} of aqueous solutions at certain temperature (e.g., 298.15K) (http://www.aim.env.uea.ac.uk/aim/model3/model3a.php)[6]. For ammonium sulphate and sodium chloride, the solute molality b, as well as ρ_{sol} and σ_{lv}, can be calculated on-line by prescribing the bulk solution water activity (a_w as a series of RH ranging from 0.1 to 0.9999).

a_w parameterization model I (KD). Kreidenweis et al.[8] proposed an empirical parameterization, which relates the water activity of AS and NaCl solution with g_f determined in hygroscopic growth measurements:

$$g_f = \frac{D_{sol}}{D_s} = \left(1 + \left(\sum_q k_q a_w^{q-1}\right)\frac{a_w}{1-a_w}\right)^{\frac{1}{3}} \tag{17}$$

The coefficients k_q for AS are k_1 = 2.42848, k_2 = -3.85261, and k_3 = 1.88159. The respective coefficients for NaCl are k_1 = 5.78874, k_2 = -8.38172, and k_3 = 3.9265. The method was demonstrated by HTDMA measured hygroscopic data for dry AS and NaCl particles with spherical equivalent mobility diameter of 100 nm. By comparing with observational data and AIM modelling results, this parameterized relationship was validated for the AS and NaCl solution droplets at $x_s < 0.3$ and $x_s < 0.13$, respectively[8]. Thus, with each single measurement of g_f of dry AS and NaCl particle, one could retrieve the corresponding a_w by iteratively solving supplementary equation (17).

a_w parameterization model II (TM). Tang and Munkelwitz[7,38] presented parameterizations of water activity a_w in aqueous solution droplet of AS and NaCl derived from single particle experiments by the EDB (Electrodynamic Balance method). The parameterization of a_w is expressed as polynomial fit functions of solute mass fraction (x_s):

$$a_w = 1 + \sum_q A_q (100 \cdot x_s)^q \tag{18}$$

The polynomial coefficients A_q for AS at 298 K are $A_1 = -2.175\times10^{-3}$, $A_2 = 3.113\times10^{-5}$, $A_3 = -2.336\times10^{-6}$, and $A_4 = 1.412\times10^{-8}$. The respective coefficients for NaCl are $A_1 = -6.366\times10^{-3}$, $A_2 = 8.624\times10^{-5}$, $A_3 = -1.158\times10^{-5}$, and $A_4 = 1.518\times10^{-7}$. According to the EDB experiments[7,38], the validated range of this parameterization method is $x_s < 0.78$ for AS and $x_s < 0.45$ for NaCl. It is used by Biskos et al.[21,23] to predict their measured hygroscopic growth curve (against RH) of nano-sized AS and NaCl particles.

σ_{lv} parameterization model I (SP). Seinfeld and Pandis[9] proposed the following parameterization for the liquid-vapour interfacial energy of aqueous salt solution droplets:

$$\sigma_{\mathrm{lv}} = \sigma_w + k_s c_{\mathrm{s}} \tag{19}$$

in which k_{s} = 2.17×10^{-3} N m^{-1} L mol^{-1} for AS and k_{s} = 1.62×10^{-3} N m^{-1} L mol^{-1} for NaCl. c_{s} is the molarity of the solution, which is defined as the amount of substance divided by the volume of the solution in units of mol L^{-1}. σ_{w} is the surface density of pure water. In the present study, σ_{w} is treated as temperature-dependent and is described by[9]:

$$\sigma_{\mathrm{w}} = 0.0761 - 1.55\times10^{-4}(T - 273.15) \tag{20}$$

σ_{lv} parameterization model II (PK). Pruppacher and Klett[5] proposed another parameterization for σ_{lv} of aqueous AS and NaCl solutions as

$$\sigma_{\mathrm{lv}} = 0.072 + \frac{2.34\times10^{-2}\cdot x_s}{1-x_s} \qquad \text{for AS} \tag{21}$$

$$\sigma_{\mathrm{lv}} = 0.072 + \frac{2.9\times10^{-2}\cdot x_s}{1-x_s} \qquad \text{for NaCl} \tag{22}$$

of which the validated range is $x_{\mathrm{s}} < 0.78$ for AS and $x_{\mathrm{s}} < 0.45$ for NaCl[24]. It is used by Biskos et al.[21,23] to predict their measured hygroscopic growth curve (against RH) of nano-sized AS and NaCl particles.

Solution density ρ_{sol}. In the present study, the concentration-dependent solution densities of AS and NaCl are parameterized based on the EDB measurements[7,38] as polynomial fit functions of solute mass fraction x_{s}:

$$\rho_{sol} = \rho_w + \sum_q d_q (100\cdot x_s)^q \tag{23}$$

The polynomial coefficients d_{q} for AS at 298 K are d_1 = 5.92, d_2 = -5.036×10^{-3}, and d_3 = 1.024×10^{-5}. The respective coefficients for NaCl are d_1 = 7.41, d_2 = -3.741×10^{-2}, d_3 = 2.252×10^{-3},

and d_4 = -2.06×10^{-5}. Here ρ_w is the density of pure water in kg m^{-3}. In the present study, a temperature-dependent ρ_w is used[5]:

$$\rho_w = \frac{\sum_q A_q \cdot (T - 273.15)^{q-1}}{1 + B \cdot (T - 273.15)} \quad (24)$$

where T is the temperature in K. The coefficients A_q are A_1 = 999.8396, A_2 = 18.224944, A_3 = -7.92221×10^{-3}, A_4 = -55.44846×10^{-6}, A_5 = 149.7562×10^{-9}, and A_6 = -393.2952×10^{-12}. The coefficient B is 18.159725×10^{-3}.

Since we are dealing with highly supersaturated AS solution, we adopted a parameterization suggested by Clegg and Wexler[39] in which the extended solution density was constrained by the density of molten salt. The density of molten AS is estimated to be ~1.61×10^3 kg m^{-3} at 298.15K. It shows general good agreement with non-constrained density parameterization[38,39] when x_s is below ~0.45, but when x_s approaches 1.0, the deviations increases to up to 3-4%[39]. We also found that the smallest growth factor of 6-nm AS particles observed during dehydration prior to crystallization (g_f ~1.041, corresponding to a AS:H_2O molar ratio of 7:1)[21] cannot be explained by the non-constrained AS solution density. The minimum ρ_{sol} required to explain such small g_f is ~1.57×10^3 kg m^{-3}, falling into the range of the ρ_{sol} constraint by the molten salt properties.

Disjoining pressure. Among earlier efforts to explain the deliquescence/efflorescence, Djikaev et al.[40] and Shchekin et al.[41] introduced a "disjoining pressure" in their models. The effect of "disjoining pressure" makes the existence of partially dissolved solute possible, which will result in a continuous deliquescence as RH increases. To explain the continuous deliquescence is one of the major goals of introducing the disjoining pressure in Djikaev et al.[40]. In other words, a prompt deliquescence would suggest that the solute is fully dissolved and the effect of disjoining pressure is negligible[40]. This is exactly the case for the HTDMA data that we were using. Therefore, the disjoining pressure was not considered in our model simulation. The negligibility of the disjoining pressure was further confirmed by the good agreement between the observation and our model prediction.

Supplementary Note 4. Melting temperature of atmospheric biogenic secondary organic aerosols

Cappa and Wilson[42] indicate that the secondary organic aerosol (SOA) particles with median volume weighted diameter of ~100 nm formed through α-pinene ozonolysis might be in a solid amorphous state rather than liquid. On the other hand, bouncing experiments clearly show that particle bounce decreases with decreasing particle size in sub 30 nm size range, suggesting a different phase state of larger (> 30 nm, solid-like) and smaller (17-30 nm, liquid-like) particles[43,44]. In order to explore the size effect on the biogenic SOA particles (such as pine-derived SOA) and to estimate the relevant critical diameter range that is able to depress its melting temperature down to the ambient conditions (~298 K) according to the T_{bulk}-$D_{\mathrm{s,c}}$ relationship (Fig. 5), we need to know the general melting temperature range of such biogenic SOA particles. The relatively low-volatility oxidation products of pinenes include multifunctionalized acids, such as pionic acid, pinic acid, 1,2,3-propane-tricarboxylic acid, 1,2,4-butane-tricarboxylic acid, 3-methyl-1,2,3-butane-tricarboxylic acid, etc. Koop et al.[45] summarized the melting temperature of some of these oxidation products in the range of ~378-439 K. They also estimated that the glass transition temperature (T_g) of pinene-derived SOA in the range of ~240-300 K, and the ratio between glass transition and melting temperature is about 0.7. So, the melting temperature of pinene-derived SOA can be calculated to be about 340-430 K, accordingly. Combining these two ranges, we consider the melting temperature range of pinene-derived SOA to be in the range of 340 K to 440 K, as a conservative estimation.

Supplementary Discussion. Heuristic viewpoint concerning the phase transition

In this section, we will discuss the size dependence of phase transition concerning the following aspects: (1) the relationship between different compounds, (2) the relationship between different phase transition processes (melting, glass transition, etc.), and (3) an outlook for future work in these directions.

(1) Relationship between different compounds

The similarity in the T_{bulk}-$D_{s,c}$ relations suggests the following supplementary equation (25), a close relationship between σ_{sl} (the interfacial energy at the solid-liquid interface) and ΔH (enthalpy of phase transition)

$$\frac{\sigma_{sl} V_m}{\Delta H} \approx \text{constant} \tag{25}$$

where V_m is the molar volume of solid. The deduction of supplementary equation (25) is as follows. According to the Gibbs-Thomson equation, the depression of phase transition (melting) temperature ΔT of particles of diameter D is

$$\Delta T = T_{bulk} - T(D) = T_{bulk} \frac{4\sigma_{sl} V_m}{\Delta H D} \tag{26}$$

$$1 - \frac{T(D)}{T_{bulk}} = \frac{4\sigma_{sl} V_m}{\Delta H D} \tag{27}$$

$$f(T_{bulk}) = \left(\frac{4\sigma_{sl} V_m}{\Delta H}\right) D^{-1} \tag{28}$$

As shown in Fig. 5, data pairs (T_{bulk}, $D_{s,c}^{-1}$) of different compounds/systems (salts, organics and SOA) converge onto a compact correlation. It means that

$$D_i \approx D_j \Rightarrow T_{bulk,i} \approx T_{bulk,j} \tag{29}$$

According to supplementary equation (28), the validation of supplementary equation (29) would require $\frac{\sigma_{sl} V_m}{\Delta H} \approx$ constant, suggesting a close relationship between the interfacial energy and the enthalpy of phase transition. This relationship, supplementary equation (25), reminds us the Turnbull empirical equation[18]

$$\frac{\sigma_{sl} V_m^{2/3}}{\Delta H} \approx \text{constant} \tag{30}$$

which differs slightly from our equation by a cube root of V_m. For comparison, we reanalyse the data of Turnbull[18] along with corresponding data for molten salts (Supplementary Fig. 7a). We find these data also fit well to our equation (Supplementary Fig. 7b). Note that Supplementary Fig. 7 contains a variety of metals (17 metals such as mercury, gold, silver, lead, etc) and molten salts, and our results contain organics and different water-salt mixed systems. These facts give us confidence that we might be able to generalize supplementary equation (25) for a large number of compounds in both melting and dissolution processes.

(2) Relationship between different phase transition processes

Beside dissolution and melting processes, size dependence has been found for other phase transition processes, such as glass transition, nucleation and spinodal decomposition[46] (Supplementary Fig. 4 and references therein). There are few studies providing size dependent data from different phase transition processes for the same compound. The freezing and melting temperatures of water in Supplementary Fig. 4 are the only data we found in literature. The measured T_m (melting temperature) and simulated T_f (freezing temperature) show very similar size dependence. The reason for the similarity is still not clear, but it has been found that the characteristic temperatures for different phase transition processes often show simple relations. For example, $T_g \approx 0.7 * T_m$[45] and $T_f \approx 0.82 * T_m$[18], despite of different theories involved (thermodynamics equilibrium for melting and nucleation theory for freezing). If such relations hold at each size range, i.e.,

$$T_g(D) = 0.7 * T_m(D) \quad \text{and} \quad T_f(D) = 0.82 * T_m(D) \tag{31}$$

substituting it into supplementary equation (27) would give

$$1-\frac{T_g(D)}{T_{\mathrm{bulk},g}}=\frac{4\sigma_{\mathrm{sl}}V_m}{\Delta HD} \qquad \text{and} \qquad 1-\frac{T_f(D)}{T_{\mathrm{bulk},f}}=\frac{4\sigma_{\mathrm{sl}}V_m}{\Delta HD} \qquad (32)$$

which shows similar size dependence as for the melting temperature. Supplementary equation (32) would support the similarity of T_{bulk}-$D_{\mathrm{s,c}}$ relationship between different phase transition processes.

(3) Outlook and future work

Size dependence could provide further insight into the underlying mechanisms of phase transition and should be pursued more extensively. In the past, size dependent phase transitions have been studied for different compounds. Here, we would expect a deeper insight from size dependence measurements of different phase transition processes for the same substance. Such measurements would provide another dimension to validate, improve and possibly reconcile the various existing theories for different phase transition phenomena (such as melting, nucleation, spinodal decomposition, glass transition, dissolution, etc.).

It is known that the interaction with a substrate can significantly change the size dependence of phase transitions for investigated samples. To properly account for or to avoid interaction with a substrate is essential for the comparison and synthetic studies aforementioned. The substrate-free methods based on the tandem DMA as demonstrated in this study provides a new experimental tool in this direction. Molecular dynamics simulations will be a key modelling tool in reconciling the mechanisms leading to size dependence of different phase transition processes.

Supplementary References

1 Archer, D. G. Thermodynamic Properties of the NaCl+H_2O System. II. Thermodynamic Properties of NaCl(aq), NaCl-$2H_2O$(cr), and Phase Equilibria. *J. Phys. Chem. Ref. Data* **21**, 793-829 (1992).

2 Chan, C. K., Liang, Z., Zheng, J., Clegg, S. L. & Brimblecombe, P. Thermodynamic Properties of Aqueous Aerosols to High Supersaturation: I - Measurements of Water Activity of the System $Na^{+}-Cl^{-}-NO_3^{-}-SO_4^{2-}-H_2O$ at ~ 298.15 K. *Aerosol Sci. Tech.* **27**, 324-344 (1997).

3 Clegg, S. L., Brimblecombe, P., Liang, Z. & Chan, C. K. Thermodynamic Properties of Aqueous Aerosols to High Supersaturation: II—A Model of the System $Na^{+}-Cl^{-}-NO^{-3}-SO_4^{2-}-H_2O$ at 298.15 K. *Aerosol Sci. Tech.* **27**, 345-366 (1997).

4 Tang, I. N., Munkelwitz, H. R. & Wang, N. Water activity measurements with single suspended droplets: The NaCl-H2O and KCl-H2O systems. *J. Colloid Interf. Sci.* **114**, 409-415 (1986).

5 Pruppacher, H. R. & Klett, J. D. *Microphysics of clouds and precipitation*. (Kluwer Academic Publishers, 1997).

6 Clegg, S. L., Brimblecombe, P. & Wexler, A. S. Thermodynamic Model of the System $H^{+}-NH_4^{+}-Na^{+}-SO_4^{2-}-NO_3^{-}-Cl^{-}-H_2O$ at 298.15 K. *J. Phys. Chem. A* **102**, 2155-2171 (1998).

7 Tang, I. N. Chemical and size effects of hygroscopic aerosols on light scattering coefficients. *J. Geophys. Res-Atmos.* **101**, 19245-19250 (1996).

8 Kreidenweis, S. M. *et al.* Water activity and activation diameters from hygroscopicity data - Part I: Theory and application to inorganic salts. *Atmos. Chem. Phys.* **5**, 1357-1370 (2005).

9 Seinfeld, J. H. & Pandis, S. N. *Atmospheric Chemistry and Physics, from Air Pollution to Climate Change*. (John Wiley, 2006).

10 Couchman, P. R. & Jesser, W. A. Thermodynamic theory of size dependence of melting temperature in metals. *Nature* **269**, 481-483 (1977).

11 Pan, D., Liu, L.-M., Slater, B., Michaelides, A. & Wang, E. Melting the Ice: On the Relation between Melting Temperature and Size for Nanoscale Ice Crystals. *ACS Nano* **5**, 4562-4569 (2011).

12 Buffat, P. & Borel, J. P. Size effect on the melting temperature of gold particles. *Phys. Rev. A* **13**, 2287-2298 (1976).

13 Morishige, K. & Kawano, K. Freezing and Melting of Methyl Chloride in a Single Cylindrical Pore: Anomalous Pore-Size Dependence of Phase-Transition Temperature. *J. Phys. Chem. B* **103**, 7906-7910 (1999).

14 Morishige, K. & Kawano, K. Freezing and Melting of Nitrogen, Carbon Monoxide, and Krypton in a Single Cylindrical Pore. *J. Phys. Chem. B* **104**, 2894-2900 (2000).

15 Manka, A. *et al.* Freezing water in no-man's land. *Phys. Chem. Chem. Phys.* **14**, 4505-4516 (2012).

16 Li, T., Donadio, D. & Galli, G. Ice nucleation at the nanoscale probes no man's land of water. *Nat Commun* **4**, 1887 (2013).

17 Forrest, J. A. & Mattsson, J. Reductions of the glass transition temperature in thin polymer films: Probing the length scale of cooperative dynamics. *Phys. Rev. E* **61**, R53-R56 (2000).

18 Turnbull, D. Formation of Crystal Nuclei in Liquid Metals. *J. Appl. Phys.* **21**, 1022-1028 (1950).

19 Buckle, E. R. & Ubbelohde, A. R. Studies on the Freezing of Pure Liquids. I. Critical Supercooling in Molten Alkali Halides. *P. Roy. Soc. Lond. A Mat.* **259**, 325-340 (1960).

20 Buckle, E. R. & Ubbelohde, A. R. Studies on the Freezing of Pure Liquids. III. Homogeneous Nucleation in Molten Alkali Halides. *P. Roy. Soc. Lond. A Mat.* **261**, 197-206 (1961).

21 Biskos, G., Paulsen, D., Russell, L. M., Buseck, P. R. & Martin, S. T. Prompt deliquescence and efflorescence of aerosol nanoparticles. *Atmos. Chem. Phys.* **6**, 4633-4642 (2006).

22 DeCarlo, P. F., Slowik, J. G., Worsnop, D. R., Davidovits, P. & Jimenez, J. L. Particle Morphology and Density Characterization by Combined Mobility and Aerodynamic Diameter Measurements. Part 1: Theory. *Aerosol Sci. Tech.* **38**, 1185-1205 (2004).

23 Biskos, G., Russell, L. M., Buseck, P. R. & Martin, S. T. Nanosize effect on the hygroscopic growth factor of aerosol particles. *Geophys. Res. Lett.* **33**, L07801 (2006).

24 Dahneke, B. E. Slip correction factors for nonspherical bodies—III the form of the general law. *J. Aerosol Sci.* **4**, 163-170 (1973).

25 Erdemir, D., Lee, A. Y. & Myerson, A. S. Nucleation of Crystals from Solution: Classical and Two-Step Models. *Accounts Chem. Res.* **42**, 621-629 (2009).

26 Bahadur, R. & Russell, L. M. Effect of Surface Tension from MD Simulations on Size-Dependent Deliquescence of NaCl Nanoparticles. *Aerosol Sci. Tech.* **42**, 369-376 (2008).

27 Brechtel, F. J. & Kreidenweis, S. M. Predicting Particle Critical Supersaturation from Hygroscopic Growth Measurements in the Humidified TDMA. Part I: Theory and Sensitivity Studies. *J. Atmos. Sci.* **57**, 1854-1871 (2000).

28 Gilbert, B., Huang, F., Zhang, H., Waychunas, G. A. & Banfield, J. F. Nanoparticles: Strained and Stiff. *Science* **305**, 651-654 (2004).

29 Cohen, M. D., Flagan, R. C. & Seinfeld, J. H. Studies of concentrated electrolyte solutions using the electrodynamic balance. 3. Solute nucleation. *J. Phys. Chem.* **91**, 4583-4590 (1987).

30 Richardson, C. B. & Snyder, T. D. A Study of Heterogeneous Nucleation in Aqueous Solutions. *Langmuir* **10**, 2462-2465 (1994).

31 Ostwald, W. Über die vermeintliche Isomerie des roten und gelben Quecksilbersoxyds und die Oberflächenspannung fester Körper. *Z. Phys. Chem.* **34**, 495-503 (1900).

32 Freundlich, H. *Kapillarchemie. Eine Darstellung der Chemie der Kolloide und verwandter Gebiete*. (Akademische Verlagsgesellschaft, 1909).

33 Chen, J.-P. Theory of Deliquescence and Modified Köhler Curves. *J. Atmos. Sci.* **51**, 3505-3516 (1994).

34 Gao, Y., Chen, S. B. & Yu, L. E. Efflorescence Relative Humidity for Ammonium Sulfate Particles. *J. Phys. Chem. A* **110**, 7602-7608 (2006).

35 Gao, Y., Chen, S. B. & Yu, L. E. Efflorescence relative humidity of airborne sodium chloride particles: A theoretical investigation. *Atmos. Environ.* **41**, 2019-2023 (2007).

36 Wu, W. & Nancollas, G. H. Determination of interfacial tension from crystallization and dissolution data: a comparison with other methods. *Adv. Colloid Interfac.* **79**, 229-279 (1999).

37 Dutcher, C. S., Ge, X., Wexler, A. S. & Clegg, S. L. An Isotherm-Based Thermodynamic Model of Multicomponent Aqueous Solutions, Applicable Over the Entire Concentration Range. *J. Phys. Chem. A* **117**, 3198-3213 (2013).

38 Tang, I. N. & Munkelwitz, H. R. Water activities, densities, and refractive indices of aqueous sulfates and sodium nitrate droplets of atmospheric importance. *J. Geophys. Res-Atmos.* **99**, 18801-18808 (1994).

39 Clegg, S. L. & Wexler, A. S. Densities and Apparent Molar Volumes of Atmospherically Important Electrolyte Solutions. 1. The Solutes H_2SO_4, HNO_3, HCl, Na_2SO_4, $NaNO_3$, NaCl, $(NH_4)_2SO_4$, NH_4NO_3, and NH_4Cl from 0 to 50 °C, Including Extrapolations to Very Low Temperature and to the Pure Liquid State, and $NaHSO_4$, NaOH, and NH_3 at 25 °C. *J. Phys. Chem. A* **115**, 3393-3460 (2011).

40 Djikaev, Y. S. *et al.* Theory of size dependent deliquescence of nanoparticles: Relation to heterogeneous nucleation and comparison with experiments. *J. Phys. Chem. B* **105**, 7708-7722 (2001).

41 Shchekin, A. K., Shabaev, I. V. & Rusanov, A. I. Thermodynamics of droplet formation around a soluble condensation nucleus in the atmosphere of a solvent vapor. *J. Chem. Phys.* **129**, 214111 (2008).

42 Cappa, C. D. & Wilson, K. R. Evolution of organic aerosol mass spectra upon heating: implications for OA phase and partitioning behavior. *Atmos. Chem. Phys.* **11**, 1895-1911 (2011).

43 Virtanen, A. *et al*. An amorphous solid state of biogenic secondary organic aerosol particles. *Nature* **467**, 824-827 (2010).

44 Virtanen, A. *et al*. Bounce behavior of freshly nucleated biogenic secondary organic aerosol particles. *Atmos. Chem. Phys.* **11**, 8759-8766 (2011).

45 Koop, T., Bookhold, J., Shiraiwa, M. & Poschl, U. Glass transition and phase state of organic compounds: dependency on molecular properties and implications for secondary organic aerosols in the atmosphere. *Phys. Chem. Chem. Phys.* **13**, 19238-19255 (2011).

46 Burch, D. & Bazant, M. Z. Size-Dependent Spinodal and Miscibility Gaps for Intercalation in Nanoparticles. *Nano Lett.* **9**, 3795-3800 (2009).
